# Supplementary material for: RUNX3 regulates cell cycle-dependent chromatin dynamics by functioning as a pioneer factor of the restriction-point
Source: Nat Commun. 2019 Apr 23;10:1897. doi: 10.1038/s41467-019-09810-w (PMC6479060; doi:10.1038/s41467-019-09810-w)
Supplement: Supplementary file 1 — Supplementary Information [file 41467_2019_9810_MOESM1_ESM.pdf]

RUNX3 regulates cell cycle-dependent chromatin dynamics by functioning as a pioneer factor of the restriction-point

Lee et al.

Supplementary Fig. 1

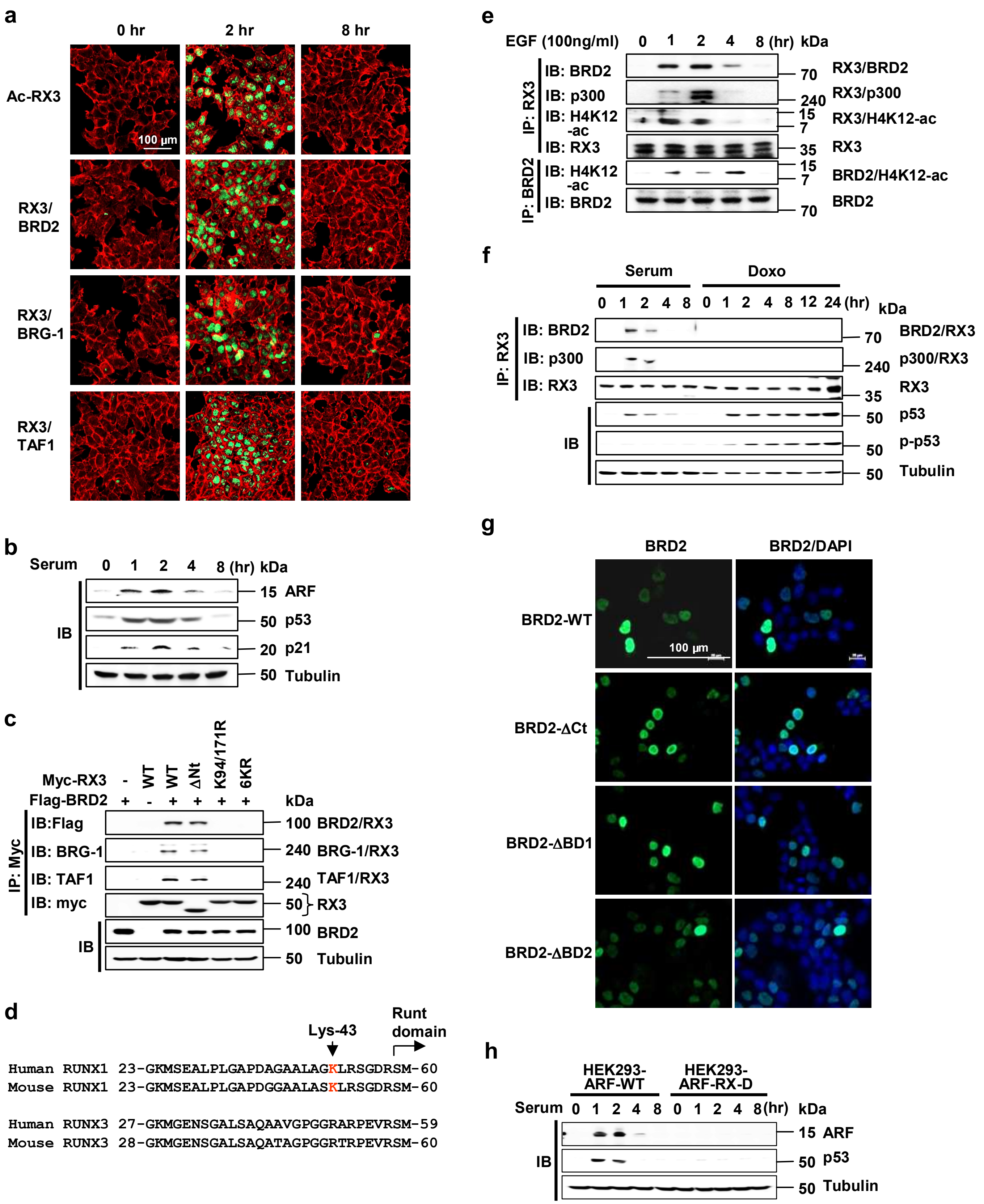

Supplementary Fig. 1 RUNX3 interacts with the SWI/SNF complex and TFIID complex through BRD2

a HEK293 cells were serum-starved for 24 hr, and then stimulated with 10% serum. Proximity ligation assay (PLA) was performed at the indicated times after serum stimulation. Green fluorescence: association of the indicated proteins or modification of the indicated protein. F-actin was stained (red) to visualize the cytoplasmic compartment.

b HEK293 cells were serum-starved for 24 hr, and then stimulated with 10% serum. Expression of *ARF*, *p53*, and *p21* was measured by immunoblotting (IB) at the indicated times after serum stimulation.

c HEK293 cells were transfected with *Myc-RUNX3-WT*, *Myc-RUNX3-ΔNt* (lacking N-terminal aa 1–54 region), *Myc-RUNX3-K94/171R* , *Myc-RUNX3-6KR*, or *Flag-BRD2-WT*, as indicated. Myc-RX3-6KR mutant harbors K (Lys) to R (Arg) mutations in all 6 K residues within Runt domain of RUNX3. The Myc-RUNX3-K94/171R mutant, which does not interact with BRD2, harbors K94R and K171R mutations within the Runt domain of RUNX3. Cells were serum-starved for 24 hr, and then stimulated with 10% serum. Cells were harvested 2 hr after stimulation, and the interactions of RUNX3 mutants with BRD2, BRG-1 (SWI/SNF), and TAF1 (TFIID) were measured by IP and IB. BRG-1 and TAF1 interacted with RUNX3 only when the RUNX3–BRD2 complex was formed.

d Amino acid sequence alignment of the coding region for human/mouse RUNX1 and RUNX3. The Lys-43 residue, which is critical for the direct interaction between RUNX1 and TAF1, is absent in RUNX3.

e HEK293 cells were serum-starved for 24 hr, and then stimulated with EGF (100 ng/ml). Cells were harvested at the indicated time points, and the time-dependent interactions between RUNX3, BRD2, p300, and H4K12-ac and the levels of acetylated RUNX3 (RX3-ac) were measured by IP and IB.

f HEK293 cells were serum-starved for 24 hr, and then stimulated with 10% serum or doxorubicin (1 μM). Cells were harvested at the indicated time points, and the time-dependent interactions between RUNX3, BRD2, p300, and the levels of p53 and phosphorylated p53 at Ser-15 (p-p53) were measured by IP and/or IB.

g HEK293 cells were transfected with *Flag-BRD2-WT*, *Flag-BRD2-ΔCt*, *Flag-BRD2-ΔBD1*, or *Flag-BRD2-ΔBD2*, as indicated. Subcellular localization of the expressed proteins was analyzed by immunofluorescence staining (green=BRD mutants; blue=DAPI).

h HEK293-ARF-WT and HEK293-ARF-RX-D cells were serum-starved for 24 hr. The cells were then treated with 10% serum, and the time-dependent expression of *ARF* and *p53* was measured by IB.

Supplementary Fig. 2

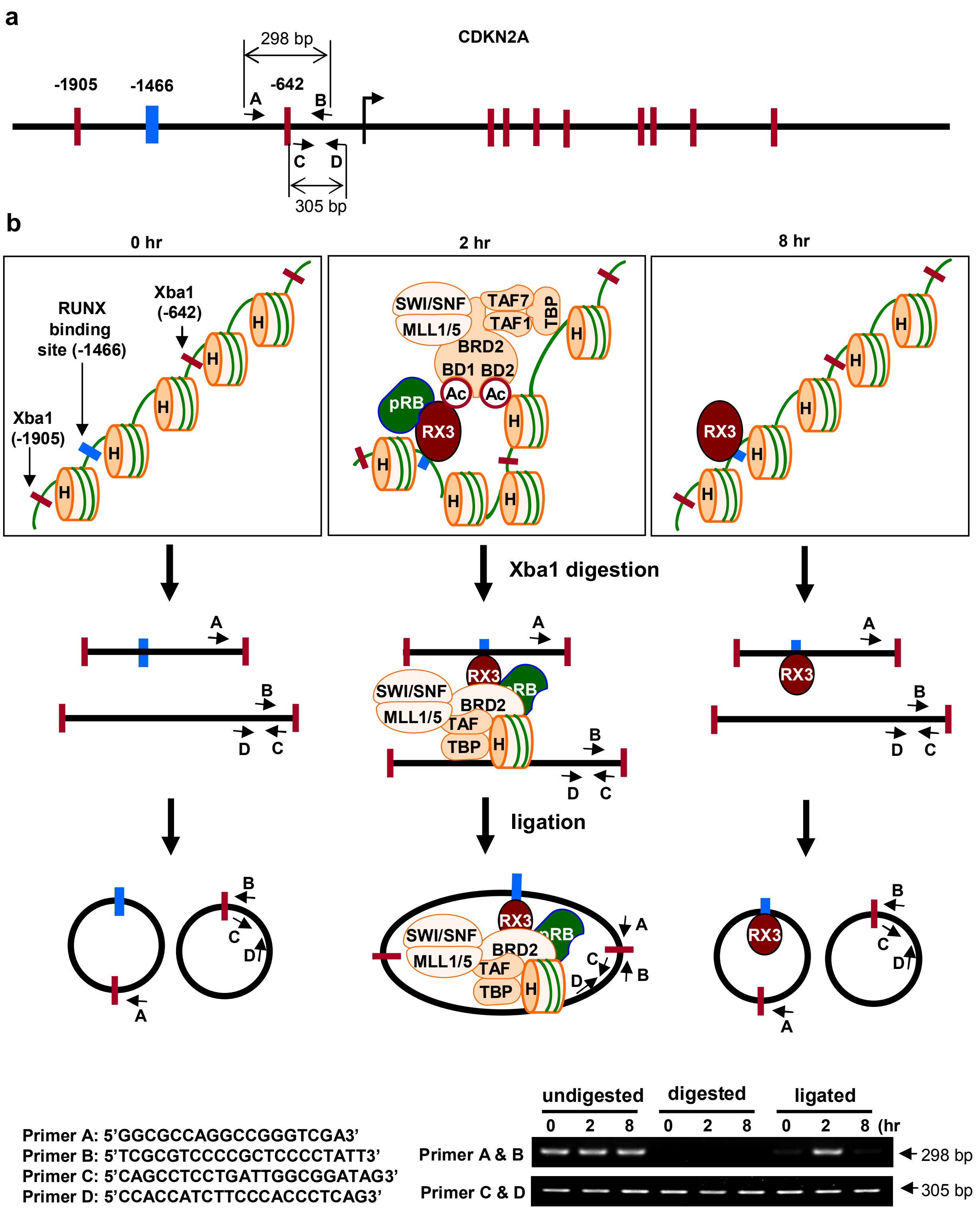

Supplementary Fig. 2 Schematic illustration showing the structure of *ARF* locus and 3C assay

a Structure of *ARF* locus showing RUNX binding site (blue bar), *Xba*1 sites (red bar) and primers (arrows).

b Predicted structure of *ARF* locus showing at the indicated time points (0, 2, 8 hr) after serum stimulation. 3C analysis was carried out as described in the Method Details. To detect chromatin looping, two combinations of primer pairs were used for semi-quantified PCR following enzymatic digestion and identified by gel electrophoresis (undigested : undigested genomic DNA , digested : digested genomic DNA by *Xba*1 enzyme, ligated : ligated genomic DNA after *Xba*1 digestion.)

Supplementary Fig. 3

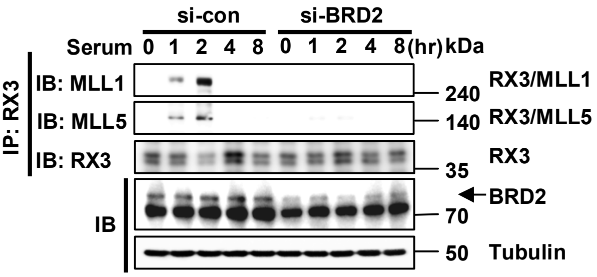

Supplementary Fig. 3. BRD2 mediates the interactions between MLL1/5 and RUNX3. HEK293 cells were treated with control (si-con) or BRD2-specific siRNA (si-BRD2), serum-starved for 24 hr, and then stimulated with 10% serum for the indicated periods of time. The time-dependent interactions between MLL1/5 and RUNX3 were monitored by IP and IB.

Supplementary Fig. 4

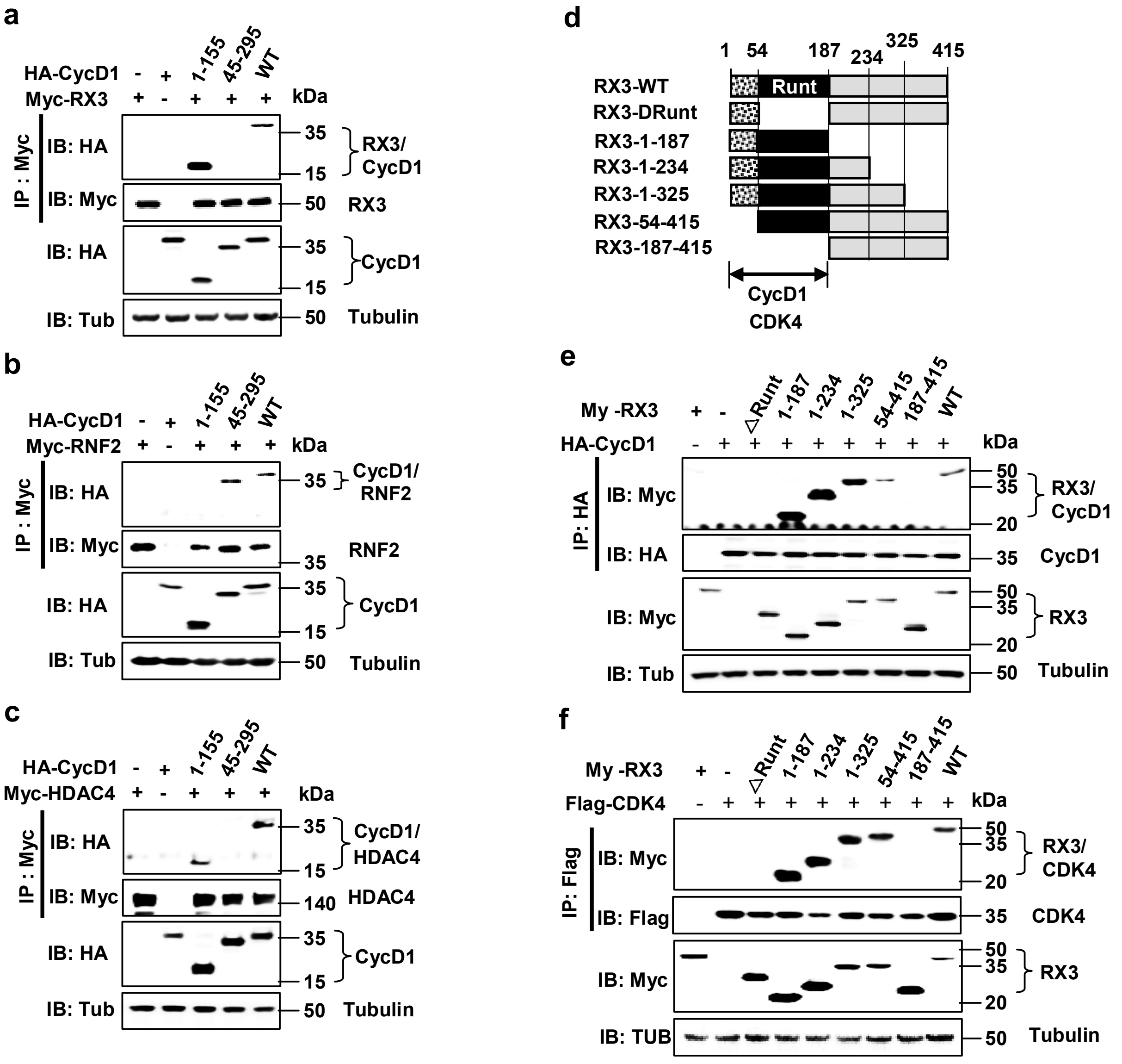

Supplementary Fig. 4 Mapping of the regions for RUNX3-Cyclin D1 and RUNX3-CDK4 interactions

a-c Myc-RUNX3, Myc-RNF2, or Myc-HDAC4 was co-expressed with HA-Cyclin D1(WT), HA-Cyclin D1(1–155), and HA-Cyclin D1(45–295) in HEK293 cells. The interactions of RUNX3, RNF2, and HDAC4 with Cyclin D1 were measured by IP and IB 4 hr after serum stimulation.

d Serial deletion mutants of Myc-RX3 were co-expressed with HA-Cyclin D1(WT) or Flag-CDK4 in HEK293 cells. The regions of RUNX3 required for the interaction with Cyclin D1 and CDK4 are indicated.

e-f The interactions between RUNX3, Cyclin D1 and CDK4 were measured by IP and IB 4 hr after serum stimulation.

Supplementary Fig. 5

a

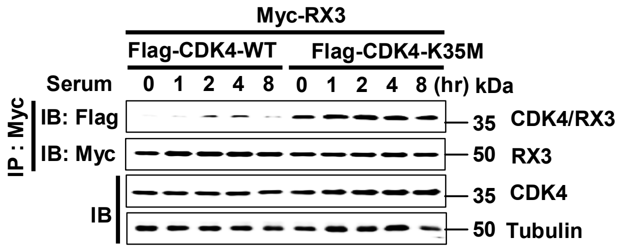

b

RUNX3: GGDRSPTRMLAS (S-356)
RUNX1: GGERSPPRILPP (S-397)
RUNX2: GGDRSPSRMLPP (S-451)

c

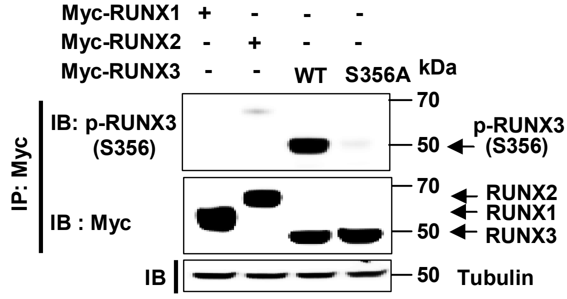

Supplementary Fig. 5 Generation of antiserum (anti-p-RUNX3-S356) recognizing phosphorylated RUNX3 at Ser-356.
a HEK293 cells were transfected with Myc-RUNX3 and Flag-CDK4-WT or Flag-CDK4-K35M (kinase inactive CDK4 mutant). Cells were serum-starved for 24 hr, stimulated with 10% serum, and harvested at the indicated time points. Time-dependent formation of the CDK4–RUNX3 complex was measured by IP and IB.
b Comparison of peptide sequence adjacent to the CDK4-dependent phosphorylation site of RUNX family members. RUNX3 peptide phosphorylated at Ser-356 was used to raise rabbit anti-serum.
c HEK293 cells were transfected with Myc-RUNX1, Myc-RUNX2, Myc-RUNX3 and Myc-RUNX3-S356A (RUNX3 mutant lacking the CDK4-dependent phosphorylation site) . At 2 hr after serum stimulation, cells were harvested, and RUNX3 phosphorylation at Ser-356 was analyzed by IP with anti-Myc antibody followed by IB with antiserum (anti-p-RUNX3-S356).

Supplementary Fig. 6

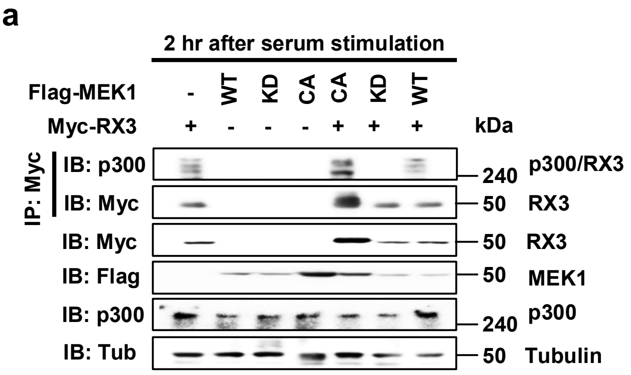

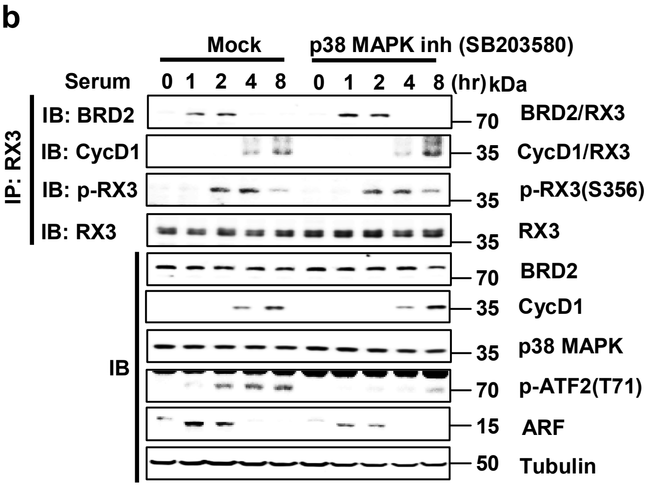

Supplementary Fig. 6. MEK signaling stimulates interactions between RUNX3, p300, and BRD2.

a HEK293 cells were transfected with *Flag-MEK1-WT*, *Flag-MEK1-CA* (constitutively active form of MEK1), *Flag-MEK1-KD* (kinase dead form of MEK1), and *Myc-RUNX3-WT* as indicated. Cells were serum-starved for 24 hr, and then stimulated with 10% serum. Cells were harvested 2 hr after stimulation, and the interaction between RUNX3 and p300 was monitored by IP and IB.

b HEK293 cells were treated with p38 MAPK inhibitor (SB203580, 1 mM), serum-starved for 24 hr, and then stimulated with serum for the indicated durations. Time-dependent interactions of BRD2–RUNX3 and CyclinD1–RUNX3, as well as phosphorylation of RUNX3 at Ser-356, were monitored by IP and IB. Time-dependent phosphorylation of ATF2 at Thr-71 was measured as a control to confirm inhibition of p38 MAPK.

Supplementary Fig. 7

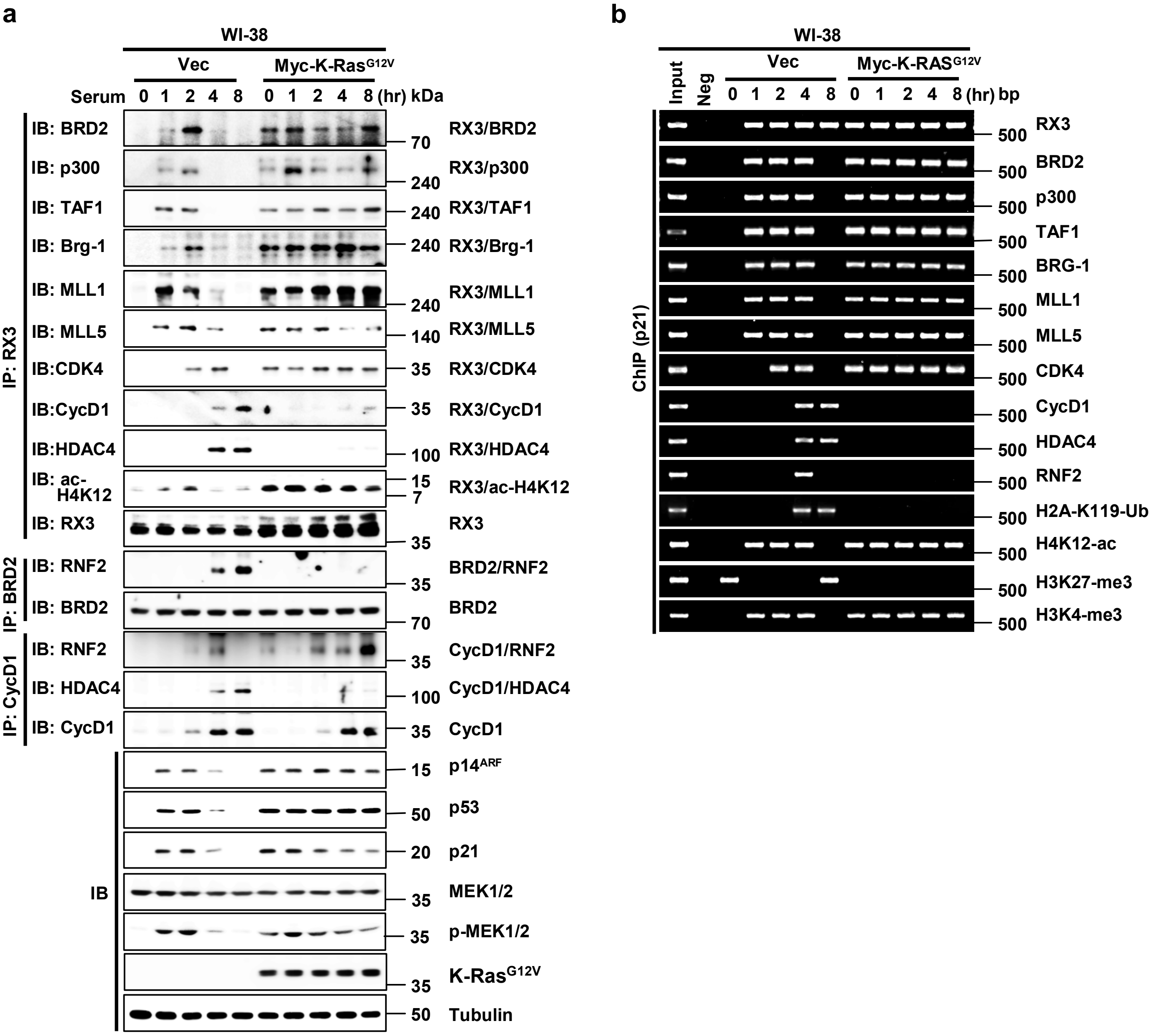

Supplementary Fig. 7. The R-point transition in WI-38 cells.

a WI-38 cells were transfected with empty vector (Vec) or Myc-K-RAS^{G12V}, serum-starved for 24 hr, stimulated with 10% serum, and harvested at the indicated time points. The time-dependent interactions among the components of Rpa-RX3-AC, Rpa-RX3-TR, and Rpa-RX3-RE were measured by IP and IB. Expression levels of ARF, p53, p21, and Myc-K-Ras^{G12V} were measured by IB.

b WI-38 cells were serum-starved for 24 hr, and then stimulated with 10% serum. Binding of the components of Rpa-RX3-AC, Rpa-RX3-TR, and Rpa-RX3-RE to the *p21* promoter and histone marks (H4K12-ac, H3K27-me3, H3K4-me3, and H2A-K119-Ubi) at the locus were measured by ChIP at the indicated time points. One-thirtieth volumes of each lysate were PCR-amplified as input samples.

Supplementary Fig. 8

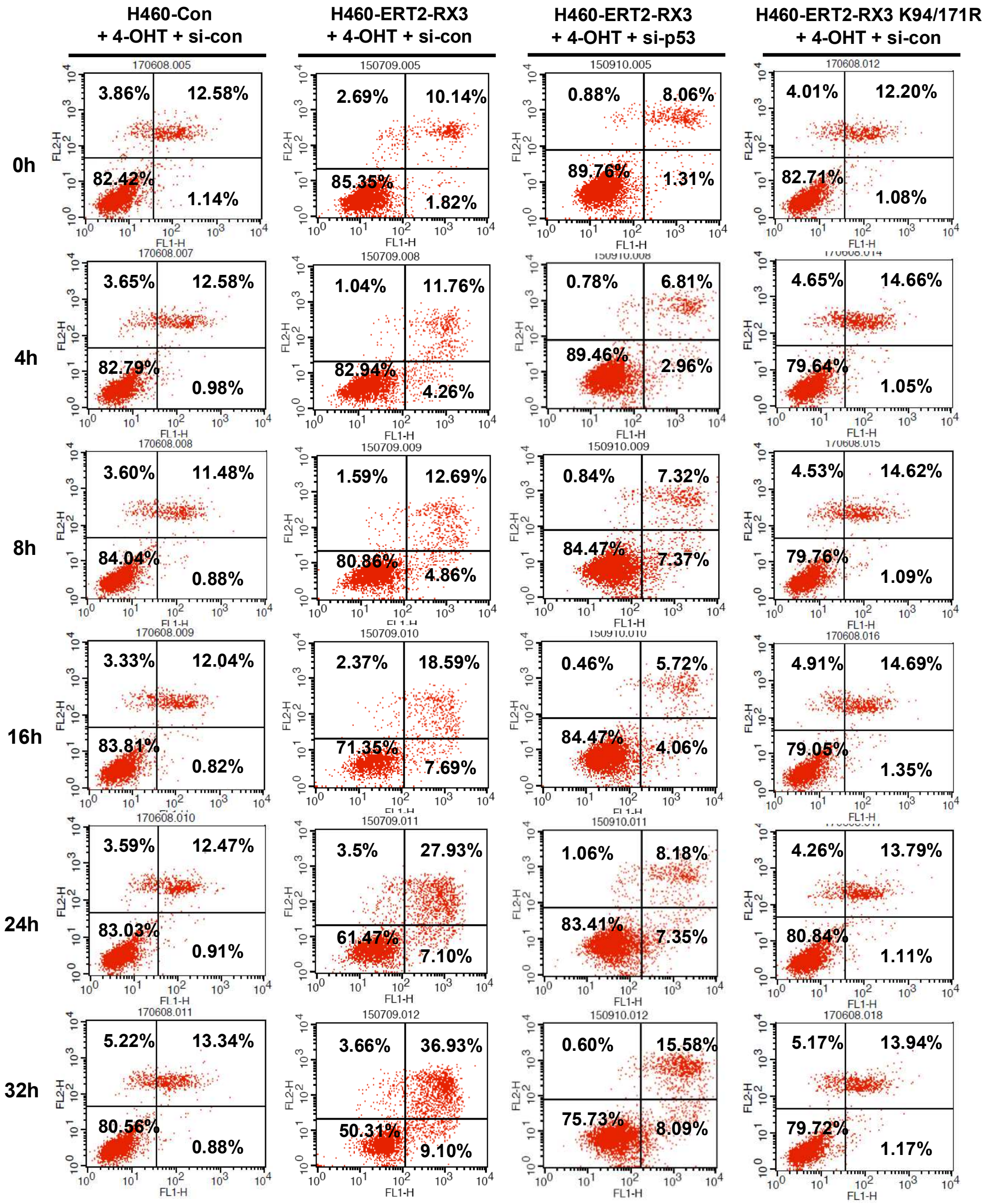

Supplementary Fig. 8 Rpa-RX3-AC defends against oncogenic K-Ras–dependent lung cancer in a cell culture model

H460, H460-ERT2-RUNX3 and H460-ERT2-RUNX3-K94/171R cells were treated with control siRNA (si-con) or p53-specific siRNA (si-p53), serum-starved for 24 hr, and then stimulated with serum/4-OHT for the indicated times. Apoptotic cells were detected by flow cytometry after Annexin V–FITC/PI staining. The abscissa and ordinate represent the fluorescence intensities of Annexin V–FITC and PI, respectively. The percentages of early apoptotic cells (Annexin V–FITC-positive/PI-negative; lower right quadrant) and late apoptotic or necrotic cells (Annexin V–FITC-positive/PI-positive; upper right quadrant) are shown.

Supplementary Fig. 9

a

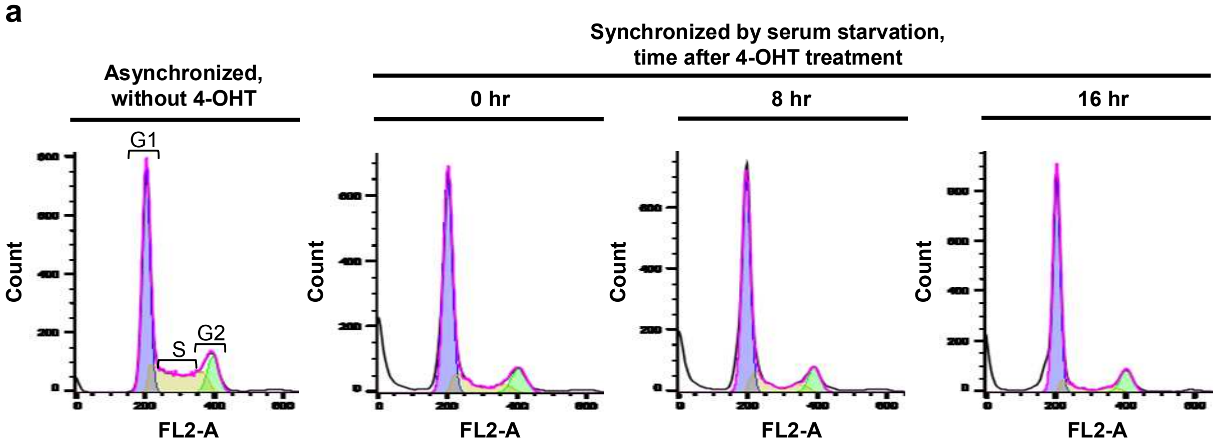

b

	Asynchronized	Synchronized, time after 4-OHT treatment		
	Con	0 hr	8 hr	16 hr
G1	48.9%	49.7%	51.5%	55.3%
S	28.4%	13.1%	12.6%	9.33%
G2	11.5%	8.75%	8.18%	9.17%

c

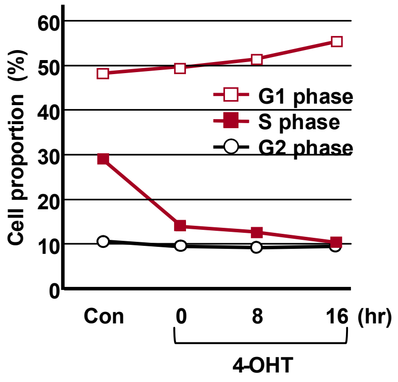

Supplementary Fig. 9. Analysis of cell-cycle stage in H460-ER-RX3 cells after 4-OHT treatment.

a H460-ER-RX3 cells were treated with 4-OHT, and populations of the cells were analyzed by FACS at various cell-cycle stages.

b Populations of H460-ER-RX3 cells after 4-OHT treatment are summarized.

c Changes in the cell-cycle stage populations of H460-ER-RX3 cells after 4-OHT treatment.

Supplementary Fig. 10

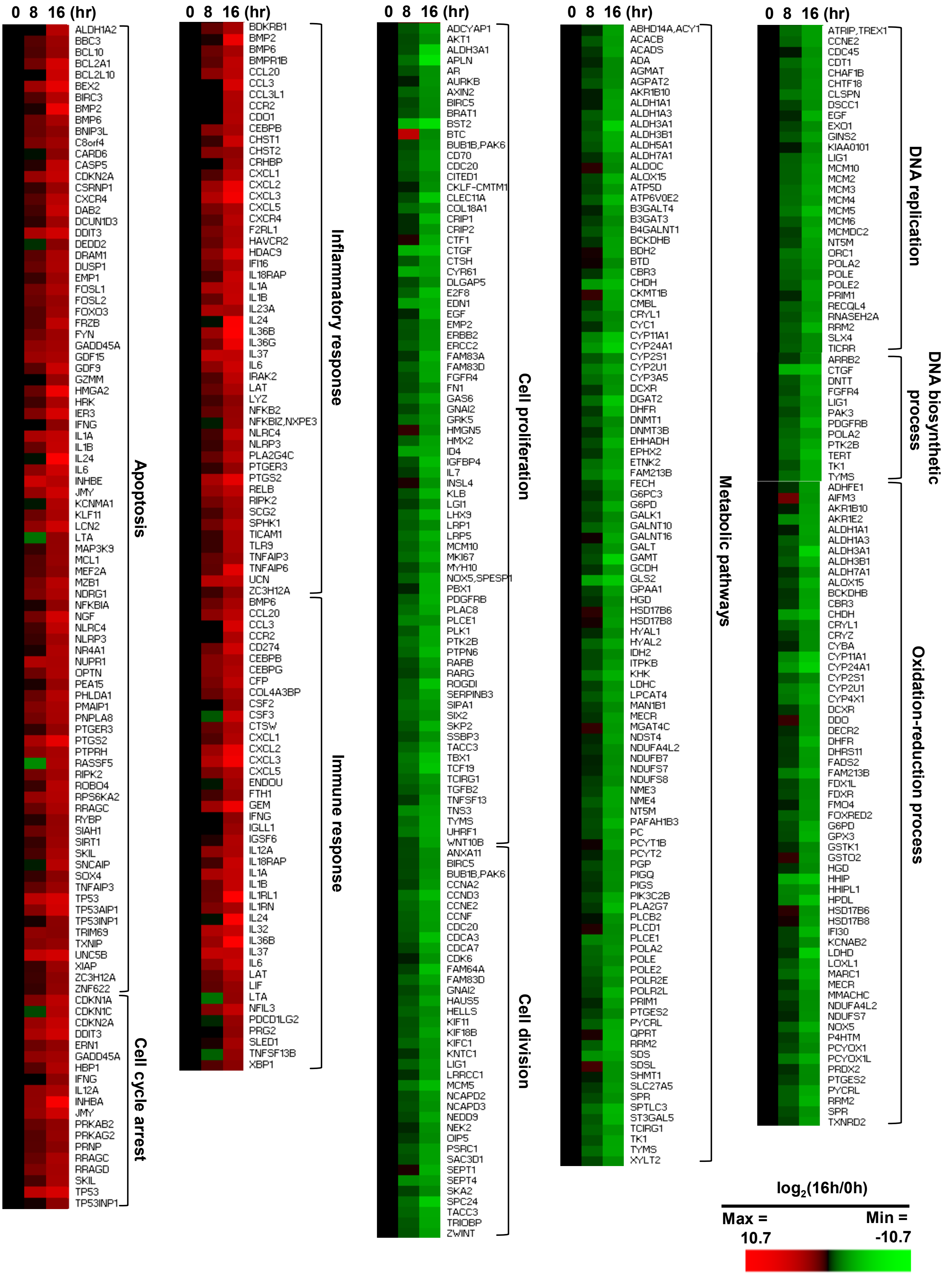

Supplementary Fig. 10 Genes up- or down-regulated by RUNX3 in H460 lung cancer cells

Heatmap showing genes up- or down-regulated by RUNX3 after 10% serum/1μM 4-OHT stimulation in H460-ERT2-RUNX3 cells. The major signaling categories of the upregulated genes include apoptosis, cell-cycle arrest, inflammatory response, and immune response. The major signaling categories of the downregulated genes include cell proliferation, cell division, metabolic pathways, DNA replication, DNA biosynthetic process, and oxidation–reduction process. FPKM-normalized values of each gene were converted to their log2 values to generate the heatmap.
