# Supplementary material for: RUNX3 regulates cell cycle-dependent chromatin dynamics by functioning as a pioneer factor of the restriction-point
Source: Nat Commun. 2019 Apr 23;10:1897. doi: 10.1038/s41467-019-09810-w (PMC6479060; doi:10.1038/s41467-019-09810-w)
Supplement: Supplementary file 3 — Reporting Summary [file 41467_2019_9810_MOESM3_ESM.pdf]

Corresponding author(s): Suk-Chul Bae

Last updated by author(s): Feb 28, 2019

Reporting Summary

Nature Research wishes to improve the reproducibility of the work that we publish. This form provides structure for consistency and transparency in reporting. For further information on Nature Research policies, see Authors & Referees and the Editorial Policy Checklist.

Statistics

For all statistical analyses, confirm that the following items are present in the figure legend, table legend, main text, or Methods section.

n/a	Confirmed	
☐	☒	The exact sample size (n) for each experimental group/condition, given as a discrete number and unit of measurement
☐	☒	A statement on whether measurements were taken from distinct samples or whether the same sample was measured repeatedly
☐	☒	The statistical test(s) used AND whether they are one- or two-sided<br>*Only common tests should be described solely by name; describe more complex techniques in the Methods section.*
☐	☒	A description of all covariates tested
☐	☒	A description of any assumptions or corrections, such as tests of normality and adjustment for multiple comparisons
☐	☒	A full description of the statistical parameters including central tendency (e.g. means) or other basic estimates (e.g. regression coefficient) AND variation (e.g. standard deviation) or associated estimates of uncertainty (e.g. confidence intervals)
☐	☒	For null hypothesis testing, the test statistic (e.g. F, t, r) with confidence intervals, effect sizes, degrees of freedom and P value noted<br>*Give P values as exact values whenever suitable.*
☐	☒	For Bayesian analysis, information on the choice of priors and Markov chain Monte Carlo settings
☐	☒	For hierarchical and complex designs, identification of the appropriate level for tests and full reporting of outcomes
☐	☒	Estimates of effect sizes (e.g. Cohen's d, Pearson's r), indicating how they were calculated

Our web collection on statistics for biologists contains articles on many of the points above.

Software and code

Policy information about availability of computer code

Data collection	Imaging Software for Microscopy (Carl Zeiss, Zen 2012 SP2(blue edition)), ImageQuant TL (General image analysis software, GE Healthcare Life Sciences, Cat# 29000605), STAR software (http://star.mit.edu/index.html), Cufflinks software (http://cole-trapnell-lab.github.io/cufflinks/install)
Data analysis	PermutMatrix (Clustering tool, http://www.atgc-montpellier.fr/permutmatrix), DAVID Bioinformatics Resources 6.8 (https://david.ncifcrf.gov/home.jsp), FlowJo (https://www.flowjo.com)

For manuscripts utilizing custom algorithms or software that are central to the research but not yet described in published literature, software must be made available to editors/reviewers. We strongly encourage code deposition in a community repository (e.g. GitHub). See the Nature Research guidelines for submitting code & software for further information.

Data

Policy information about availability of data

All manuscripts must include a data availability statement. This statement should provide the following information, where applicable:

- Accession codes, unique identifiers, or web links for publicly available datasets
- A list of figures that have associated raw data
- A description of any restrictions on data availability

All raw data are available in "Figures-Lee et al-Original Data" and "Supplementary Figures-Original Data" files.

Field-specific reporting

Please select the one below that is the best fit for your research. If you are not sure, read the appropriate sections before making your selection.

☒ Life sciences ☐ Behavioural & social sciences ☐ Ecological, evolutionary & environmental sciences

For a reference copy of the document with all sections, see nature.com/documents/nr-reporting-summary-flat.pdf

Life sciences study design

All studies must disclose on these points even when the disclosure is negative.

Sample size	Not applicable
Data exclusions	No data was excluded
Replication	All results were replicated
Randomization	Not applicable
Blinding	Not applicable

Reporting for specific materials, systems and methods

We require information from authors about some types of materials, experimental systems and methods used in many studies. Here, indicate whether each material, system or method listed is relevant to your study. If you are not sure if a list item applies to your research, read the appropriate section before selecting a response.

Materials & experimental systems

n/a	Involved in the study	
☐	☒	Antibodies
☐	☒	Eukaryotic cell lines
☒	☐	Palaeontology
☒	☐	Animals and other organisms
☒	☐	Human research participants
☒	☐	Clinical data

Methods

n/a	Involved in the study	
☒	☐	ChIP-seq
☐	☒	Flow cytometry
☒	☐	MRI-based neuroimaging

Antibodies

Antibodies used

Antibodies targeting Cyclin D1 (Cat# sc-20044), CDK4 (Cat# sc-260), HDAC4 (Cat# sc-11418), p-c-Jun (Cat# SC-822), p-ATF (Cat# SC-8398), p110 (Cat# SC-7174), p300 (Cat# sc-584), p53 (Cat# sc-126), p21 (Cat# sc-397), p14(Cat# sc-8340, Cat# sc-53640), ERK1 (Cat# SC-94), ERK1/2 (Cat# SC-135900), E2F1 (Cat# sc-137059), TAF1 (Cat# sc-735), TAF7 (Cat# sc-292282), TBP (Cat# sc-421), BRG-1 (Cat# sc-17796, Cat# sc-10768), and BAF155 (Cat# sc-10756) were obtained from Santa Cruz Biotechnology (Dallas, TX, USA).
Antibodies targeting H2AK119-ub (Cat# 8240S), H3K412-ac (Cat# 2591S), H3K27-me3 (Cat# 9733S), H3K4-me3 (Cat# 9751S), RNF2 (Cat# 5694S), BMI1 (Cat# 6964S), EZH2 (Cat# 5246S), phospho-pRB(Ser-795) (Cat# 9301S), phospho-ERK1/2 (Cat# 9101S), JNK (Cat# 92525), S6K (Cat# 2708S), p-S6K (Cat# 9234S), p38 MAPK (Cat# 9212S) and Acetylated Lys (Cat# 9441L) were obtained from Cell Signaling Technology (Danvers, MA, USA).
Antibodies targeting RUNX3(5G4) (Cat# ab40278), p16 (Cat# ab108349) and EED (Cat# ab4469) were obtained from Abcam (Cambridge, UK).
Antibodies targeting HA (12CA5; Cat# 11 666 606 001, Sigma, MO, USA), FLAG (M2; Cat# F1804, Sigma, MO, USA), Myc (9E10; Cat# sc-40, Santa Cruz Biotechnology), BRD2 (M01; Cat# H00006046-M01, Abnova, Taipei City, Taiwan), pRB (Cat# 554136, BD Biosciences, CA, USA), p-CDK4 (Cat# PA5-64482, Invitrogen, CA, USA), MLL5 (Cat# STJ27895, St John's Laboratory, London, UK) and MLL1 (Cat# A300-374A, Bethyl Laboratories Inc., TX, USA) were used for IB and IP.
Anti RUNX3-phospho-S356 was made rabbit polyclonal anti-serum against synthetic RUNX3 peptide phosphorylated at Ser-356.

Validation

Santa Cruz Biotechnology (Dallas, TX, USA) : https://www.scbt.com
Cell Signaling Technology (Danvers, MA, USA) : https://www.cellsignal.com
Abcam (Cambridge, UK) : https://www.abcam.com
Sigma (MO, USA) : https://www.sigmaaldrich.com
Abnova (Taipei City, Taiwan) : http://www.abnova.com
BD Biosciences (CA, USA) : http://www.bdbiosciences.com
Invitrogen (CA, USA) : https://www.thermofisher.com
St John's Laboratory (London, UK) : https://www.stjohnslabs.com

Bethyl Laboratories Inc. (TX, USA) : https://www.bethyl.com

Eukaryotic cell lines

Policy information about cell lines

Cell line source(s)	HEK293 cells : Human embryonic kidney 293 cells<br>WI-38 cells : Human embryonic lung fibroblasts<br>NCI-H460 cells : Human large cell lung cancer cells
Authentication	HEK293 cells : ATCC (CRL-1573)<br>WI-38 cells : Lonza (WI-38)<br>NCI-H460 cells : ATCC (HTB-177)
Mycoplasma contamination	all of cell lines are negative for Mycoplasma contamination.
Commonly misidentified lines (See ICLAC register)	Not applicable

Flow Cytometry

Plots

Confirm that:

☒ The axis labels state the marker and fluorochrome used (e.g. CD4-FITC).

☒ The axis scales are clearly visible. Include numbers along axes only for bottom left plot of group (a 'group' is an analysis of identical markers).

☒ All plots are contour plots with outliers or pseudocolor plots.

☒ A numerical value for number of cells or percentage (with statistics) is provided.

Methodology

Sample preparation	1. Apoptosis assay by Annexin V staining<br>: Cells were harvested and processed using the FITC–Annexin V Apoptosis Detection Kit I (BD Biosciences, San Jose, CA, USA)<br>2. Cell Cycle<br>: Cells were harvested and processed using propidium iodide DNA staining.
Instrument	BD FACSCalibur machine (BD Biosciences).
Software	FlowJo (https://www.flowjo.com)
Cell population abundance	Cell population abundance : 1*10^6 cells
Gating strategy	Not applicable

☒ Tick this box to confirm that a figure exemplifying the gating strategy is provided in the Supplementary Information.
