# Supplementary material for: RUNX3 regulates cell cycle-dependent chromatin dynamics by functioning as a pioneer factor of the restriction-point
Source: Nat Commun. 2019 Apr 23;10:1897. doi: 10.1038/s41467-019-09810-w (PMC6479060; doi:10.1038/s41467-019-09810-w)
Supplement: Supplementary file 4 — Description of Additional Supplementary Files [file 41467_2019_9810_MOESM4_ESM.docx]

**Description of Additional Supplementary Files**

File Name: Supplementary Movie 1

Description: An overview of the R-point transition and R-point–associated chromatin dynamics

File Name: Supplementary Movie 2

Description: Rapid induction of apoptosis in H460 cells by RUNX3 activation.

File Name: Supplementary Data 1

Description: RNA-seq results provided in Excel file.
