# Supplementary material for: RUNX3 regulates cell cycle-dependent chromatin dynamics by functioning as a pioneer factor of the restriction-point
Source: Nat Commun. 2019 Apr 23;10:1897. doi: 10.1038/s41467-019-09810-w (PMC6479060; doi:10.1038/s41467-019-09810-w)
Supplement: Supplementary file 2 — Peer Review File [file 41467_2019_9810_MOESM2_ESM.pdf]

Reviewers' comments:

Reviewer #1 (Remarks to the Author):

The article entitled "RUNX3 regulates cell cycle-dependent chromatin dynamics by functioning as a pioneer factor of the restriction point", by J-W Lee and colleagues, depicts the importance of RUNX3 in controlling the restriction point through processive modifications of a protein complex involved in chromatin reorganization.

Overall, this is a well-written article, with convincing results demonstrating the mechanism of RUNX3 as a pioneer factor modulating chromatin environment and gene expression in a time-dependent manner. However, before any possible approval for publication, several points should be assessed:

Major points:

- It is mandatory to perform some of the IP/IB experiments under different contexts: using a different cell lines other than HEK293T as well as using a different stimulation than Serum only (like one inducing proliferation and cell cycle like EGF, and others more stress-related that should not trigger R-point).

- Similarly, to avoid context-specific biases, it is mandatory to perform some of the ChIP analyses on a different promoter than ARF only.

- the conclusion that "On the basis of these findings, we conclude that RUNX3 on its binding site in an enhancer region facilitates p300-mediated histone acetylation around the locus, juxtaposes the promoter region via formation of the RUNX3–BRD2–H4K12- ac complex, and then recruits SWI/SNF and TFIID complexes to the locus via BRD2" is a bit over-reaching. If RUNX3 is truly the first element of the complex at the promoter and further bring together the entire complex (first through p300, then BRD2 then others), an important experiment is to perform ChIP analysis using RUNX3 mutants disabling associations with the different interactors and observe the binding capacity of each element of the complex onto the promoter. For example, RUNX3 with mutated lysines K94 and K171 should still binds to promoter, brings p300 and promotes H4 acetylation but not BRD2 and further complex subunits.

Minor points:

- To disregard possible disruption of DNA/chromatin at ARF promoter after CRISPR engineering (Fig 1F), is it possible to show another TF binding ARF promoter as a control in both HEK293 ARF-WT and ARF-RX-D (RUNX1, E2F?)?

- Regarding RUNX3 and MLL proteins in complex (Fig 2), does the authors have evidence of direct interactions with RUNX3 or is it happening thanks to BRD2?

- Authors claiming "about 3 hr after mitogen stimulation" is a bit puzzling, as no single experiment has been done at 3h post stimulation. Only evidence is that this is happening between 2 and 4 h.

- In Figure 2b, authors claim that BRD2 and RUNX3 are in different complexes after 4h, but do not show IB for Cyclin D1/HDAC4 in the BRD2 IP as well as BMI1 in the RUNX3 IP.

- Authors never mentioned the possibility that HDAC4 deacetylates RUNX3, leading to dissociation with BRD2 and association with PRC1. It would be very interesting to demonstrate if this is the case.
- It would be interesting to perform similar experiment as the one using Myc-RUNX3-S356A but with a reversely constitutive phosphorylation mutant (using D or E mutation to mimic Ser-ph) and observe opposite effect.

- For experiments in Fig. 5, can the authors provide data for other pathways to demonstrate the specificity of their observations compared to parallel kinase pathways? (AKT, or p38?)
- Regarding the interesting regulation demonstrated in Fig. 5 & 6, to clearly demonstrate the specificity of this regulation, it might be important to perform similar experiment using MEK inh (trametinib) or to stimulate JNK pathway.

- Regarding schematic of Fig.6, can the authors explain how KRAS stimulating both MEK and JNK pathways fails to promote "the Rpa-RX3-AC → Rpa-RX3-TR transition" while these pathways have opposite effect in their system?

- In their H460-ERT2-RUNX3 cells model, can the authors demonstrate if the transition to the repressive complex is still happening? If so, one can imagine that combining RUNX3 expression and JNK inhibitor might have drastic apoptotic effect.
- It would be nice to have similar schematics than the ones in Fig 1 & 2 for summarizing the results of figure 3 and figure 4.

- It is unclear how the authors come up with some of the interactors they identified for the different RUNX3 complexes. Can they explain better or provide references suggesting PRC1 (as well as PRC2) interaction with Cyclin D1 and HDAC4?

- The data presented can be overwhelming at first, but the majority of the IP/IB presented are indeed repetition with subtle differences. Thus, it could be a possibility to lighten these data by selecting only the essential results in the main figures with more details into supplemental figures (for example, showing only IB for one subunit of a complex, or only one IP and its corresponding IB, etc.).

Reviewer #2 (Remarks to the Author):

Bae and co-workers used immunoprecipitation and ChIP in a breathtaking endeavor to study the formation of transcriptional activator and repressor complexes as a function of time after serum re-stimulation.

Based on the dynamics of protein-protein and promoter-protein interactions after mitogen stimulation, the authors demonstrate the consecutive formation of three distinct complexes centered around RUNX3. RUNX3 acts as a pioneer factor that binds specific target sites in closed chromatin and facilitates histone acetylation by p300. Subsequent recruitment of chromatin remodeling proteins via its binding partner BRD2 generates an activator complex (AC) accumulating 2-4 h after mitogen stimulation that also includes MLL1/5 to produce the activating histone modification H3K4-me3. This results in transcriptional activation of RUNX3 target genes as exemplified by p14ARF; after 4-8 h another complex accumulates that contains PRC1 complex members RNF2 and BMI1 and also Cyclin D1 and HDAC4 (TR complex). At 8 h the RUNX3-BRD2 interaction is disrupted and PRC1 is replaced for PRC2 (repressor complex RE). This is associated with re-appearance of the repressive histone modification H3K27-me3 and loss of the activating modification H3K4-me3 as well as loss of ARF expression. Fig. 2f conveniently summarizes this interpretation although the inclusion of pRB in the

early complexes is only justified later (Fig. 4a).

The picture that arises is quite complicated, but the authors have succeeded in presenting their data in clear figures and by lucid description in the manuscript text.

I only have some minor comments:

1. Where does Cyclin D1 come from? The text reminds the reader that Cyclin D1 is transcriptionally and translationally controlled through RAS-RAF and RAS-PI3K pathways, respectively. Apparently, the raise of CYCLIN D1 levels occurs independently of RUNX3, but it would be convenient if the authors could refer to existing literature on CCND1 regulation.

2. Related to the above: what is the rationale behind the early induction of p14ARF; why doesn't it stay repressed; why does ARF need to be opened by RUNX3, activated and then again suppressed? Is it to provide the cells with p53 levels?

3. Most intriguing but also counterintuitive is that oncogenic RAS inhibits the transition of activator to repressor complexes and hence prevents the shut-down of cell cycle arrest genes like ARF. How does this work mechanistically? The data summarized in Fig. 5f implicates the RAS pathway at three levels into the AC-TR-RE transition. So how should the opposite activity of oncogenic RAS be interpreted?
4. The maintenance of the activator complex as a consequence of oncogenic RAS is presented as a protective mechanism against oncogenic transformation as it leads to sustained ARF levels and hence sustained p53. Indeed, H460 cells carrying an oncogenic K-RAS mutation, undergo p53-dependent apoptosis upon induction of RUNX3 expression. Have the authors looked at which cell cycle phase the cells entered apoptosis? Does the maintenance of the AC complex keep cells stuck at the restriction point, as the manuscript seems to suggest. Or are cells capable of entering S-phase.

5. In relation to the above: how do the authors relate their finding to earlier findings that oncogenic RAS drives cells prematurely into S-phase, which leads to replication stress, DNA damage and p53-dependent oncogene-induced senescence/apoptosis? Would this indicate that inhibition or abrogation of the AC complex to RE complex transition does not preclude S-phase entry? This also raises the question whether this transition is really instrumental for restriction point passage. Experiments to study effects on S-phase entry may provide more insight.

Reviewer #3 (Remarks to the Author):

The authors have investigated the role of pioneer transcription factor RUNX3 in the restriction point. They found that there is a RUNX3-BRD2 complex that mediates changes in epigenetic factors through the sequential formation of two different protein complexes, affects gene expression, regulates the R point, and protects against oncogenes. The complexes include cyclin D1 and CDK4 and demonstrate a new role for this complex in cell cycle control. This interesting manuscript provides important new insights into the restriction point and how it is controlled. The data are clear and strong largely support the author's claims.

Comments

Supplementary Fig 1b: On what basis did the authors select these genes as R point genes?

Supplementary Figure 1c. What is the 6KR mutant that the authors are using here?

Supplementary Figure 1c. It is true that only when RX3 interacts with BRD2, it also interacts with TAF1, but these results may not be sufficient to conclude that interaction with BRD2 is required for TAF1 interaction as stated in the text.

Supplementary Figure 1c. Why does the RX3 mutant with 94-171 deleted run to the same size as the wild type RX3? Shouldn't it be smaller?

Fig 1e: The authors show that these deletion mutants are expressed at similar levels as wild type, but it is also important to know that the proteins encoded by these deletion mutants are localized to the nucleus similarly to wild type. The deletions could affect their nuclear localization.

Fig 1e. The authors state that the result of these experiments will tell them the temporal order of events, but all samples are taken at the 2 hour timepoint. They clearly and convincing show the dependencies of the interactions. They show that RX3 can interact with p300 even in the absence of BRD2, and that BD1 and BD2 subdomains of RX3 are required for binding to H4K12-ac. But these results are not sufficient to draw conclusions about the temporal order of binding. In particular, I don't believe these results from a single timepoint can prove that BRD2 simultaneously binds acetylated RUNX3 and acetylated H4 through BD1 and BD2.

Fig 1f. This is a very dramatic result. Can the authors rule out the possibility that other places in the genome were also targeted by this Crispr guide in addition to the region upstream of p14? Are all of these proteins properly expressed and localized in the HEK293-ARF-RX-D mutant cells? Is it possible there are other proteins besides RUNX3 that also bind to this region of the p14 promoter?

Fig 1g. pRB is present in the figure at 2 hours but the authors do not present data showing that pRB is part of this complex.

Fig 2f: Because the data points were actually taken at 2, 4 and 8 hours, it seems more appropriate for the authors to draw models for 2, 4 and 8 hours rather than 3 hours. RNF2 seems particularly important in distinguishing 2 and 4 hours. Can it be added to the model?

Line 259: The data in figure 2e do not demonstrate simultaneous recruitment to target loci.

Fig 5 and line 339-342: The authors show very convincingly that JNK is required for RUNX3 phosphorylation and for the dissociation of RUNX3 from BRD2 and CDK4. Are the authors arguing that phosphorylation of RX3 is mediated by CDK4 that has been phosphorylated by JNK but without any cyclin bound? CDKs do not have a good binding pocket for target proteins when cyclin is not bound so this model is surprising. Cyclin D1 levels may not have peaked at 2 hours, but it is still possible that the CDK molecules performing the phosphorylation are associated with cyclin D1 (or another cyclin D). Indeed, Fig 5d and 5e seem to suggest cyclin D1 itself is important for the dissociation of BRD2 and RX3.

Fig 7: H460 cells do not normally apoptose and they presumable have activated RAS and endogenous RX3. Why is it that introducing RX3 with an estrogen receptor and adding tamoxifen results in apoptosis?

Fig 7e,f: Related to above, if the ER-RX3 cells are apoptosing in response to 4 OHT treatment, but H460 cells do not normally spontaneously apoptose, this may not be the best conditions for determining the downstream targets of RUNX3.

Reviewers' comments:

Reviewer #1 (Remarks to the Author):

The article entitled "RUNX3 regulates cell cycle-dependent chromatin dynamics by functioning as a pioneer factor of the restriction point", by J-W Lee and colleagues, depicts the importance of RUNX3 in controlling the restriction point through processive modifications of a protein complex involved in chromatin reorganization.
Overall, this is a well-written article, with convincing results demonstrating the mechanism of RUNX3 as a pioneer factor modulating chromatin environment and gene expression in a time-dependent manner. However, before any possible approval for publication, several points should be assessed:

Major points:

Comment:
It is mandatory to perform some of the IP/IB experiments under different contexts: using a different cell lines other than HEK293T as well as using a different stimulation than Serum only (like one inducing proliferation and cell cycle like EGF, and others more stress-related that should not trigger R-point).

Answer:
In response to the reviewer's comment, we analyzed the association/ dissociation of Rpa-RX3-AC in an embryonic human lung fibroblast cell line (WI-38). The results revealed that association/dissociation of Rpa-RX3-AC also occurred in WI-38 cells with kinetics similar to those in HEK293 cells (i.e., at similar time points). We included these results in Supplementary Figure 8a and described them as follows: "The Rpa-RX3-AC → Rpa-RX3-TR → Rpa-RX3-RE transition at the R-point and maintenance of Rpa-RX3-AC by oncogenic K-RAS occurred not only in HEK293 cells, but also in WI-38 human embryonic lung fibroblasts (Suppl. Fig. 8a)." (page 13, lines 15-18). The results and figure legends are shown below.

Suppl. Fig. 8a

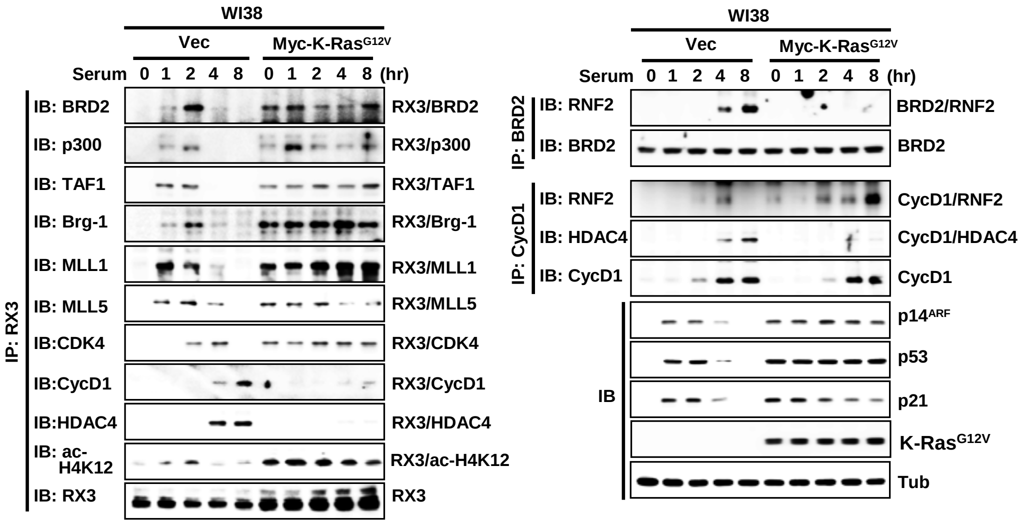

Supplementary Fig. 8. The R-point transition in WI-38 cells.

a WI-38 cells were transfected with empty vector (Vec) or Myc-K-RAS^{G12V}, serum-starved for 24 hr, stimulated with 10% serum, and harvested at the indicated time points. The time-dependent interactions among the components of Rpa-RX3-AC, Rpa-RX3-TR, and Rpa-RX3-RE were measured by IP and IB. Expression levels of ARF, p53, p21, and Myc-K-Ras^{G12V} were measured by IB.

We also analyzed the association/dissociation of Rpa-RX3-AC in response to EGF (an inducer of proliferation) or doxorubicin (an inducer of DNA damage) in HEK293 cells. Treatment with EGF induced interactions between RUNX3, BRD2, and acetylated Histone 4, and p53 was stabilized with kinetics similar to those of serum stimulation. Treatment of doxorubicin also stabilized p53, but did not induce an interaction between RUNX3 and BRD2. We included these results in Supplementary Figure 1 and described them in the manuscript as follows: "We analyzed the transient formation of RUNX3–BRD2 complex in response to EGF (an inducer of proliferation) or doxorubicin (an inducer of DNA damage). Transient formation of the RUNX3–BRD2 complex was detected 1–2 hr after treatment of EGF (Suppl. Fig. 1e), but not after treatment with doxorubicin (Suppl. Fig. 1f), although both induced p53. These results suggest that transient formation of the RUNX3–BRD2 complex is R-point–specific." (page 5, lines 25-30). The results and figure legends are shown below.

Suppl. Fig. 1e

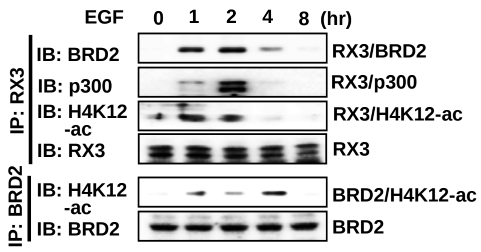

Suppl. Fig. 1f

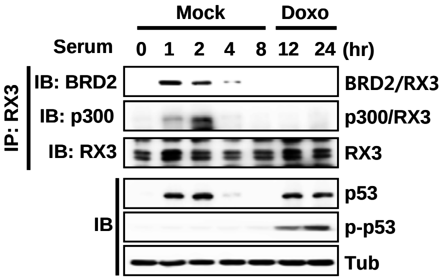

e HEK293 cells were serum-starved for 24 hr, and then stimulated with EGF (100 ng/ml). Cells were harvested at the indicated time points, and the time-dependent interactions between RUNX3, BRD2, p300, and H4K12-ac and the levels of acetylated RUNX3 (RX3-ac) were measured by IP and IB.

f HEK293 cells were serum-starved for 24 hr, and then stimulated with 10% serum or doxorubicin (1 μM). Cells were harvested at the indicated time points, and the time-dependent interactions between RUNX3, BRD2, p300, and the levels of p53 and phosphorylated p53 at Ser-15 (p-p53) were measured by IP and/or IB.

Comment:

Similarly, to avoid context-specific biases, it is mandatory to perform some of the ChIP analyses on a different promoter than ARF only.

Answer:

We previously showed that p21 expression is also transiently induced during the R-point, and that the level is maintained for a long period of time due to expression of oncogenic K-Ras (Chi, Oncogene, 2017). Therefore, we performed ChIP analyses on the p21 promoter after serum stimulation. The results were essentially the same as those for ARF. These results are now included in Supplementary Figure 8b and described in the manuscript as follows: "The results of ChIP analyses of the p21 promoter yielded essentially the same results as the analogous experiments for ARF (Suppl. Fig. 8b)." (page 13, lines 21-22). The results and figure legends are shown below.

Suppl. Fig. 8b

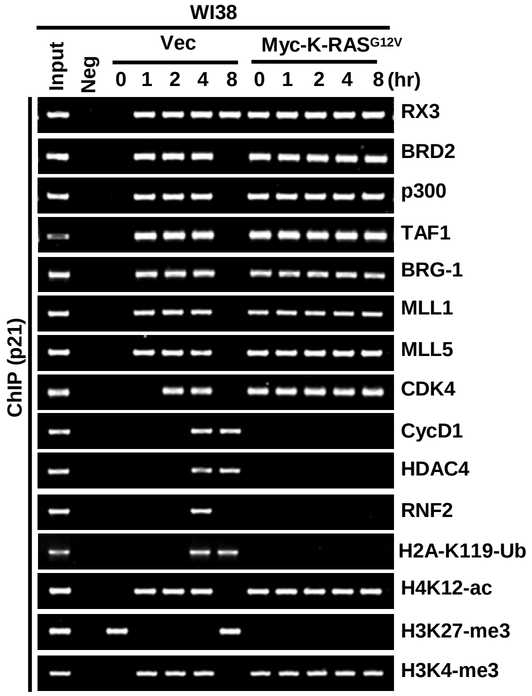

b WI-38 cells were serum-starved for 24 hr, and then stimulated with 10% serum. Binding of the components of Rpa-RX3-AC, Rpa-RX3-TR, and Rpa-RX3-RE to the *p21* promoter and histone marks (H4K12-ac, H3K27-me3, H3K4-me3, and H2A-K119-Ubi) at the locus were measured by ChIP at the indicated time points. One-thirtieth volumes of each lysate were PCR-amplified as input samples.

Comment:

The conclusion that "On the basis of these findings, we conclude that RUNX3 on its binding site in an enhancer region facilitates p300-mediated histone acetylation around the locus, juxtaposes the promoter region via formation of the RUNX3–BRD2–H4K12- ac complex, and then recruits SWI/SNF and TFIID complexes to the locus via BRD2" is a bit over-reaching. If RUNX3 is truly the first element of the complex at the promoter and further bring together the entire complex (first through p300, then BRD2 then others), an important experiment is to perform ChIP analysis using RUNX3 mutants disabling associations with the different interactors and observe the binding capacity of each element of the complex onto

the promoter. For example, RUNX3 with mutated lysines K94 and K171 should still binds to promoter, brings p300 and promotes H4 acetylation but not BRD2 and further complex subunits.

Answer:

Results addressing the reviewer's question were provided in the original manuscript (Fig. 7b and 7c). In the revised manuscript, the figure numbers were changed to Figure 8b and 8c. The results are exactly the same as the reviewer predicted: after 4-OHT treatment, ER-RUNX3-K94/171R bound the ARF locus and recruited p300, but failed to interact with BRD2 and additional subunits of the complex. Some of the results are shown below.

Fig. 8b

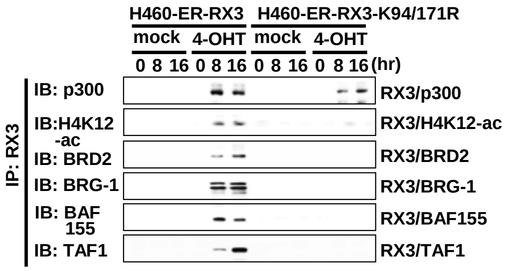

Fig. 8c

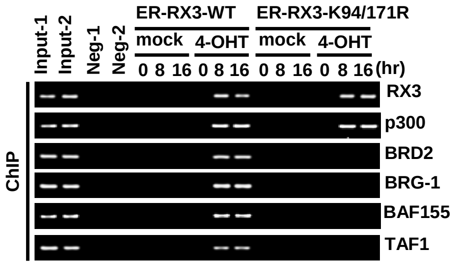

Minor points:

Comment:

To disregard possible disruption of DNA/chromatin at ARF promoter after CRISPR engineering (Fig 1F), is it possible to show another TF binding ARF promoter as a control in both HEK293 ARF-WT and ARF-RX-D (RUNX1, E2F?)?

Answer:

The ARF promoter contains four E2F-binding sites between ntd -300 and +50 from the transcription initiation site (Robertson and Jones, Mol Cell Biol. 1998; 18(11): 6457-73). To confirm that only the RUNX-binding sites were disrupted by our CRISPR engineering, we sequenced the region from ntd -2000 to +50 in HEK293-ARF-RX-D cells. The results confirmed that only the RUNX-binding site, and no other mutation, had been introduced into the ARF promoter region. We described these results in Experimental Procedures as follows: "Deletion of only the RUNX3-binding site in the

ARF promoter region was confirmed by nucleotide sequencing." (page 23, lines 13-14). We included a short nucleotide sequence illustrating this point in Figure 1f, as follows:

Fig. 1f

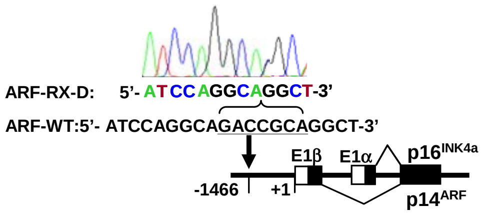

Comment:

Regarding RUNX3 and MLL proteins in complex (Fig 2), does the authors have evidence of direct interactions with RUNX3 or is it happening thanks to BRD2?

Answer:

To answer the reviewer's question, we treated cells with control or BRD2-specific siRNA and performed IP/IB analysis. We showed these results in Supplementary Figure 3 and described them in the manuscript as follows: "The interactions between RUNX3 and MLL1/5 were markedly reduced by knockdown of BRD2, confirming that the RUNX3–MLL1/5 interactions are mediated by BRD2 (Suppl. Fig. 3)." (page 7, line 36 - page 8, line 2). The results and figure legends are shown below.

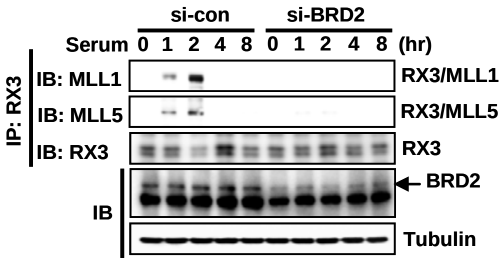

Supplementary Fig. 3. BRD2 mediates the interactions between MLL1/5 and RUNX3.

HEK293 cells were treated with control (si-con) or BRD2-specific siRNA (si-BRD2), serum-starved for 24 hr, and then stimulated with 10% serum for the indicated periods of time. The time-dependent interactions between MLL1/5 and RUNX3 were monitored by IP and IB.

Comment:

Authors claiming "about 3 hr after mitogen stimulation" is a bit puzzling, as no single experiment has been done at 3h post stimulation. Only evidence is that this is happening between 2 and 4 h.

Answer:

As the reviewer commented, our results show that complex forms between 2 and 4 hr. Therefore, we changed "3 hr after mitogen stimulation" to "between 2 and 4 hr after mitogen stimulation" (page 8, line 20).

Comment:

-In Figure 2b, authors claim that BRD2 and RUNX3 are in different complexes after 4h, but do not show IB for Cyclin D1/HDAC4 in the BRD2 IP as well as BMI1 in the RUNX3 IP.

Answer:

In response to the reviewer's comment, we performed IP/IB analysis. The new results supporting our claim are included in Figure 2b as shown below (new results are indicated with a red box).

Fig. 2b

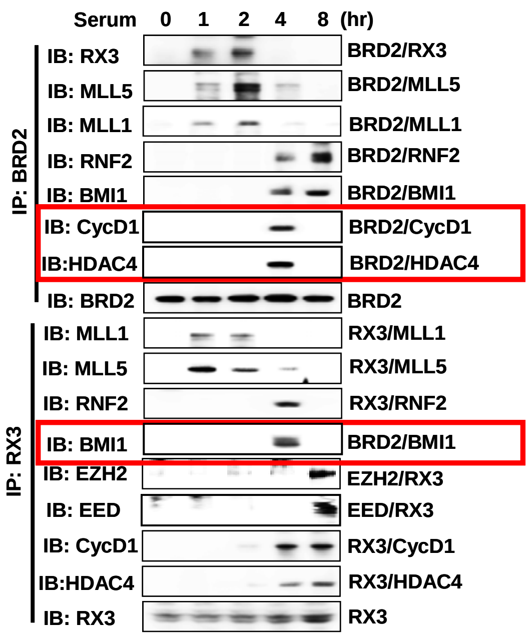

Comment:

Authors never mentioned the possibility that HDAC4 deacetylates RUNX3, leading to dissociation with BRD2 and association with PRC1. It would be very interesting to demonstrate if this is the case.

Answer:

We previously reported that p300-mediated RUNX3 acetylation is effectively removed by HDAC4 (Jin et al., J Biol Chem. 2004; 279(28): 29409-17). Thus, HDAC4 could deacetylate RUNX3 and histones within Rpa-RX3-TR. In response to the reviewer's comment, we described this interpretation in the revised manuscript as follows: "Because p300-mediated RUNX3 acetylation is effectively removed by HDAC4 (Jin et al., J Biol Chem. 2004; 279(28): 29409-17), HDAC4, a component of Rpa-RX3-TR, may play a key role in deacetylation of RUNX3, causing it to release BRD2 and other BRD2-associated proteins." (page 9, lines 21-24).

Comment:

- It would be interesting to perform similar experiment as the one using Myc-RUNX3-S356A but with a reversely constitutive phosphorylation mutant (using D or E mutation to mimic Ser-ph) and observe opposite effect.

Answer:

To answer the reviewer's comment, we constructed Myc-RUNX3-S356E, which mimics phosphorylation. IP/IB analysis revealed that Myc-RUNX3-S356E exhibited the same association/dissociation with BRD2 as Myc-RUNX3-WT, whereas, by contrast, Myc-RUNX3-S356A (a non-phosphorylatable mutant) failed to dissociate from BRD2. We included these results in Figure 4g and described them in the manuscript as follows: "However, a phosphorylation-mimic mutation of RUNX3 (Myc-RUNX3-S356E) did not affect association/dissociation of RUNX3 and BRD2 (Fig. 4g). These results suggest that RUNX3 phosphorylation at S356 is required, but not sufficient, for the release of BRD2 from RUNX3." (page 11, lines 19-22). The results and figure legends are shown below.

Fig. 4

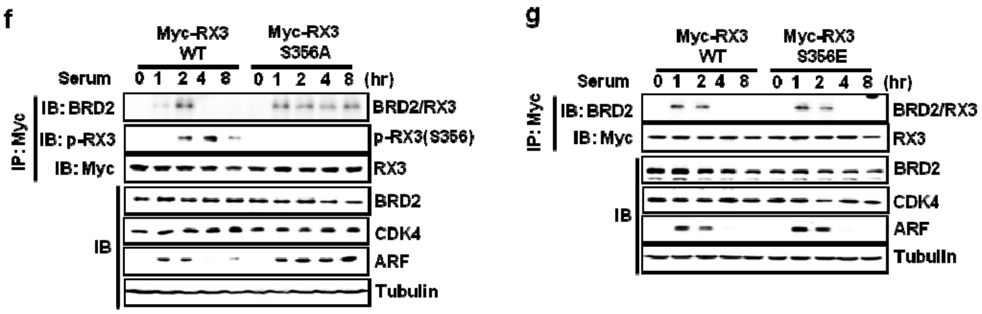

f–g HEK293 cells were transfected with Myc-RUNX3, Myc-RUNX3-S356A, or Myc-RUNX3-S356E, serum-starved for 24 hr, and then stimulated with 10% serum. Cells were harvested at the indicated time points. Time-dependent formation of the BRD2–RUNX3 complex, RUNX3 phosphorylation at Ser-356, and ARF expression were monitored by IP and IB.

Comment:

For experiments in Fig. 5, can the authors provide data for other pathways to demonstrate the specificity of their observations compared to parallel kinase pathways? (AKT, or p38?)

Answer:

PI3K signaling to mTOR is mediated by AKT. Given that PI3K and mTOR are involved in R-point transition, AKT must also be involved. To make this clear, we included AKT in the PI3K signaling pathway of Figure 5g (Fig. 6 in the revised manuscript). The modified figure is shown below.

To investigate whether p38 MAPK is involved in the R-point transition, we treated cells with p38 MAPK inhibitor and measured association/dissociation of RUNX3 and BRD2. The results revealed that SB203580 effectively inhibited phosphorylation of ATF2 at T71, a target of p38 MAPK, but did not affect association/dissociation of RUNX3 and BRD2. The results, shown below, suggest that p38 MAPK is not involved in the R-point transition. We included these results in Supplementary Figure 6 and described the results in the manuscript as follows: "Inhibition of p38 MAPK did not affect association/dissociation of RUNX3 and BRD2, suggesting that p38 MAPK is not involved in the R-point transition (Suppl. Fig. 6b)." (page 12, lines 30-32). The results and figure legends are shown below.

Fig. 6

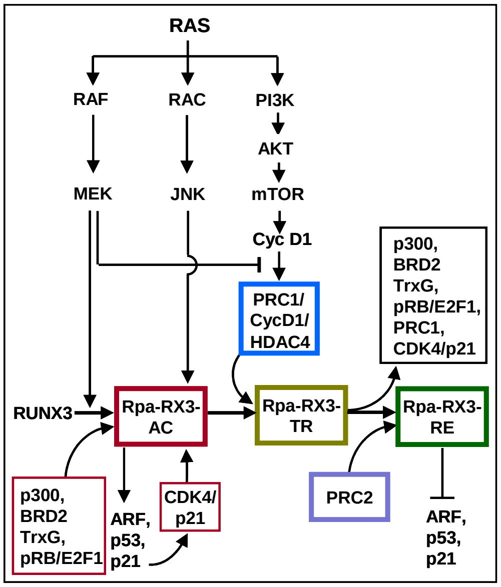

Suppl. Fig. 6b

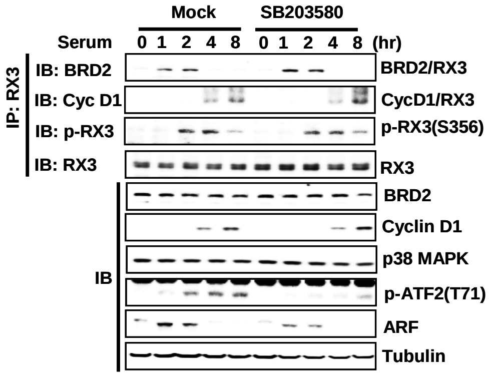

b HEK293 cells were treated with p38 MAPK inhibitor (SB203580, 1 μM), serum-starved for 24 hr, and then stimulated with serum for the indicated durations. Time-dependent interactions of BRD2–RUNX3 and CyclinD1–RUNX3, as well as phosphorylation of RUNX3 at Ser-356, were monitored by IP and IB. Time-dependent phosphorylation of ATF2 at Thr-71 was measured as a control to confirm inhibition of p38 MAPK.

Comment:

- Regarding the interesting regulation demonstrated in Fig. 5 & 6, to clearly demonstrate the specificity of this regulation, it might be important to perform similar experiment using MEK inh (trametinib) or to stimulate JNK pathway.

Answer:

In response to the reviewer's comment, we analyzed the effect of MEK inhibition in R-point regulation. Treatment with MEK1 inhibitor abolished the BRD2–RUNX3 and p300–RUNX3 interactions. Ectopic expression of MEK1-CA (constitutively activated MEK1) promoted the BRD2–RUNX3 and p300–RUNX3 interactions, whereas the MEK1-KD mutant (kinase-dead MEK1) inhibited them. We

included these results in Figure 5a and Supplementary Figure 6a and described the results in the manuscript as follows: "Treatment with MEK1 inhibitor abolished the BRD2–RUNX3 and p300–RUNX3 interactions (Fig. 5a). Consistent with this, both of these interactions were promoted by ectopic expression of constitutively activated MEK1 (MEK1-CA), but inhibited by kinase-dead MEK1 (MEK1-KD) (Suppl. Fig. 6a). These results demonstrate that the RAS–MEK signaling pathway stimulates formation of Rpa-RX3-AC." (page 11, lines 29-34). The results and figure legends are shown below.

Fig. 5a

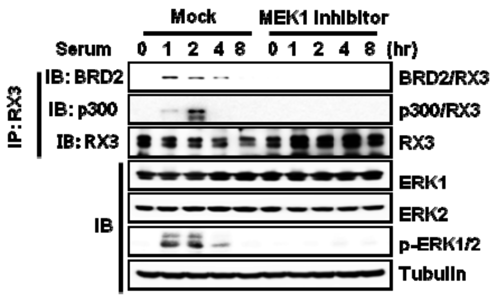

Suppl. Fig. 6a

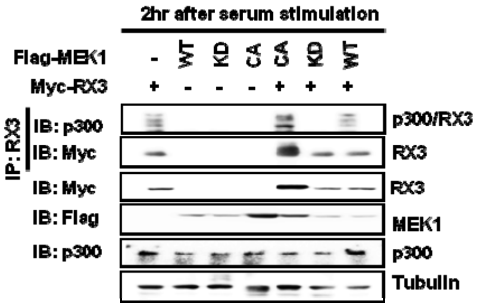

Fig. 5. Multiple signals contribute to the R-point transition.

a HEK293 cells were treated with MEK1 inhibitor (U0126, 1 μM), serum-starved for 24 hr, and then stimulated with serum for the indicated durations. Time-dependent interactions of BRD2–RUNX3 and p300–RUNX3, as well as phosphorylation of ERK1/2, were monitored by IP and/or IB.

Supplementary Fig. 6. MEK signaling stimulates interactions between RUNX3, p300, and BRD2.

a HEK293 cells were transfected with *Flag-MEK1-WT*, *Flag-MEK1-CA* (constitutively active form of MEK1), *Flag-MEK1-KD* (kinase dead form of MEK1), and *Myc-RUNX3-WT* as indicated. Cells were serum-starved for 24 hr, and then stimulated with 10% serum. Cells were harvested 2 hr after stimulation, and the interaction between RUNX3 and p300 was monitored by IP and IB.

Comment:

- Regarding schematic of Fig.6, can the authors explain how KRAS stimulating both MEK and JNK pathways fails to promote "the Rpa-RX3-AC → Rpa-RX3-TR transition" while these pathways have opposite effect in their system?

Answer:

The JNK pathway activates CDK4 after 2 hr, and the Rpa-RX3-AC → Rpa-RX3-TR transition occurs after 4 hr. Furthermore, our results show that RUNX3 phosphorylation by CDK4 at S356 is required, but not sufficient, for the Rpa-RX3-AC → Rpa-RX3-TR transition. The transition occurs after PI3K pathway–induced formation of the PRC1/CyclinD1/HDAC4 complex. Therefore, the reviewer's question could be changed to "how can K-RAS, which stimulates both the MEK and PI3K pathways, fail to promote the Rpa-RX3-AC → Rpa-RX3-TR transition?".

Mitogen-induced MEK pathway activity is maintained for only 2 hr (Fig. 4a, p-ERK1/2), and PI3K pathway–activated formation of the PRC1/CyclinD1/HDAC4 complex occurs after MEK pathway activity is downregulated. The delay of PI3K signaling leading to PRC1/CyclinD1/HDAC4 complex formation may be due to the requirement for *de novo* synthesis of CyclinD1. Our results show that oncogenic K-RAS inhibits assembly of HDAC4 into the PRC1–CyclinD1 complex (Fig. 6a, RX3/CycD1, RX3/HDAC4, RNF2/CycD1, HDAC4/CycD1). Therefore, not only PI3K pathway activation, but also downregulation of MEK activity, is required for formation of the PRC1/CyclinD1/HDAC4 complex, which plays a key role in the Rpa-RX3-AC → Rpa-RX3-TR transition. However, when the MEK activity stimulated by oncogenic K-RAS, which is not downregulated, the Rpa-RX3-AC → Rpa-RX3-TR transition cannot occur. We believe that this is one of the mechanisms by which cells measure the duration of mitogenic signal to defend against oncogenic K-RAS.

To clarify this point, we performed additional experiments demonstrating that constitutive activation of the MEK pathway inhibits the CyclinD1–HDAC4 interaction, which is critical for formation of the PRC1/CyclinD1/HDAC4 complex. We included these results and schematic demonstration in Figure 7e and 7f and described the results in the manuscript as follows: "Our results suggested that the MEK pathway stimulates formation of Rpa-RX3-AC but inhibits the Rpa-RX3-AC → Rpa-RX3-TR transition. To investigate how the MEK pathway inhibits the Rpa-RX3-AC → Rpa-RX3-TR transition, we expressed MEK1-CA and monitored the interactions between Cyclin D1, HDAC4, and RNF2. The result revealed that MEK1-CA maintained the CyclinD1–RNF2 interaction but inhibited the CyclinD1–HDAC4 interaction, indicating that

MEK1-CA inhibits formation of the PRC1/CyclinD1/HDAC4 complex (Fig. 7e). These results suggest that the failure of the Rpa-RX3-AC → Rpa-RX3-TR transition in cells expressing oncogenic *K-RAS* or oncogenic *B-RAF* is due to the failure of PRC1/CyclinD1/HDAC4 complex formation when the MEK pathway is constitutively activated. An overview of the differential regulation of the R-point by normal RAS and oncogenic RAS is provided in Figure 7f." (page 14, lines 6-16). These new results and schematic are shown below.

Fig. 7e

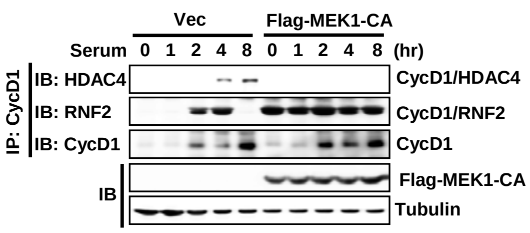

Fig. 7f

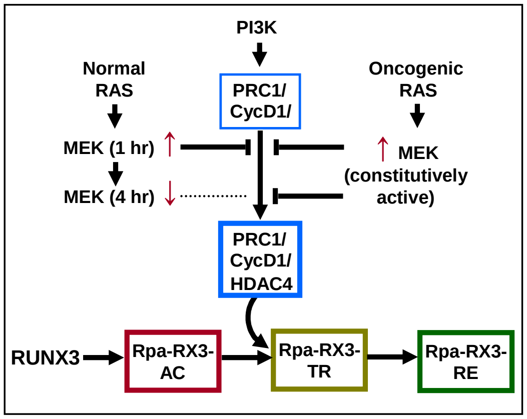

e HEK293 cells were transfected with empty vector (Vec) or Flag-MEK1-CA, serum-starved for 24 hr, stimulated with 10% serum, and harvested at the indicated time points. Time-dependent interactions of CyclinD1–HDAC4 and CyclinD1–RNF2 were monitored by IP and IB.

f Schematic illustration of differential regulation of the R-point in response to normal and oncogenic RAS. The RAS–RAF–MEK pathway inhibits formation of the PRC1/CyclinD1/HDAC4 complex, and thus inhibits the Rpa-RX3-AC → Rpa-RX3-TR transition. When the RAS–RAF–MEK pathway is activated by mitogenic stimulation, the activated pathway is downregulated after 4 hr, allowing PRC1/CyclinD1/HDAC4 complex formation, which is followed by the Rpa-RX3-AC → Rpa-RX3-TR transition. If the RAF–MEK pathway is activated by oncogenic RAS, the constitutively activated signal inhibits formation of the PRC1/CyclinD1/HDAC4 complex for a long period of time, thereby inhibiting the Rpa-RX3-AC → Rpa-RX3-TR transition.

Comment:

- In their H460-ERT2-RUNX3 cells model, can the authors demonstrate if the transition to the repressive complex is still happening? If so, one can imagine that combining RUNX3 expression and JNK inhibitor might have drastic apoptotic effect.

Answer:

We showed that the repressive complex (Rpa-RX3-RE) is not formed in cells expressing oncogenic K-RAS (Fig. 7a and 7b). Specifically, H460 cells harbor an oncogenic K-RAS mutation that inhibits formation of repressive complex (Rpa-RX3-RE). We also showed that Rpa-RX3-AC was maintained for a long period in the H460-ERT2-RUNX3 cell model, indicating that the transition to the repressive complex does not happen in K-RAS–activated cells (Fig. 8b and 8c).

Our results identifying the differential effects of RAS-activated signaling pathways on the R-point transition could contribute to the development of new strategies for treating K-RAS–activated cancers. As the reviewer commented, combining RUNX3 expression and a PI3K pathway inhibitor, which would inhibit the Rpa-RX3-AC → Rpa-RX3-TR transition, might have a dramatic therapeutic effect. However, in the interest of avoiding overinterpretation, we did not discuss this possibility in our manuscript.

Comment:

- It would be nice to have similar schematics than the ones in Fig 1 & 2 for summarizing the results of figure 3 and figure 4.

Answer: We included schematics summarizing the results in Figures 3h and 4h.
The schematics are shown below.

Fig. 3h

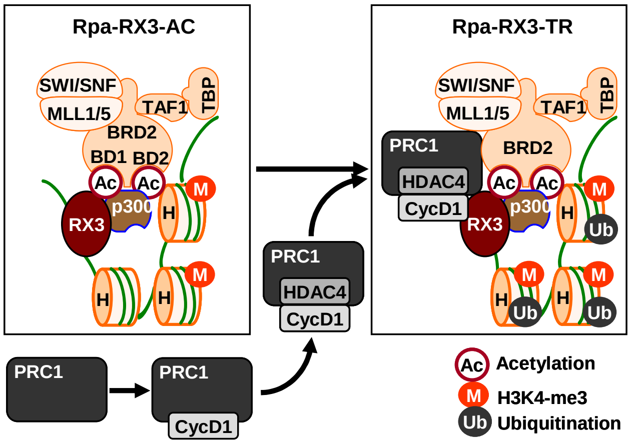

These results demonstrate that the PRC1–Cyclin D1–HDAC4 complex binds to Rpa-RX3-AC, forms Rpa-RX3-TR, and then contributes to inactivation of chromatin at target loci by deacetylating H4K12 and ubiquitinating H2A (Fig. 3h) (page 9, 29-31)

Fig. 4h

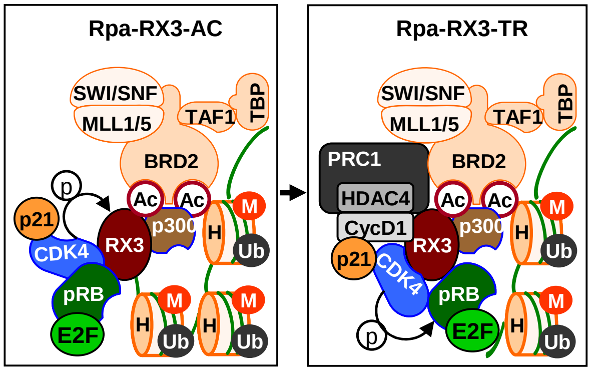

The sequence of molecular events involved in binding of CDK4 to RUNX3 (Rpa-RX3-AC), recruitment of PRC1-Cyclin D1–HDAC4 complex, and phosphorylation of RUNX3 and pRB are summarized in Fig. 4h. (page 11, lines 22-25)

Comment:

- It is unclear how the authors come up with some of the interactors they identified for the different RUNX3 complexes. Can they explain better or provide references suggesting PRC1 (as well as PRC2) interaction with Cyclin D1 and HDAC4?

Answer:

We identified some of the RUNX3-interacting proteins by yeast two-hybrid screening (MLL5 and RNF2 were identified as BRD2-interacting proteins). We were unsure why MLL5 (opening chromatin) and RNF2 (closing chromatin as a component of PRC1), which play opposite roles in chromatin dynamics, both interact with BRD2. We realized, however, that MLLs and RNF2 bound BRD2 at different time points: MLL1/5 1–2 hr after serum stimulation and RNF2 after 4 hr. We investigated how BRD2 changed its partners, and found that CDK4, CyclinD1, and HDAC4 participated in this process. Because these were our own results, no references are available regarding any of these interactions.

Comment:

- The data presented can be overwhelming at first, but the majority of the IP/IB presented are indeed repetition with subtle differences. Thus, it could be a possibility to lighten these data by selecting only the essential results in the main figures with more details into supplemental figures (for example, showing only IB for one subunit of a complex, or only one IP and its corresponding IB, etc.).

Answer:

To emphasize that the proteins interact as complexes, rather than separate proteins, we analyzed two subunits of each complex in IP/IB experiments and showed all the results in the figures.

Reviewer #2 (Remarks to the Author):

Bae and co-workers used immunoprecipitation and ChIP in a breathtaking endeavor to study the formation of transcriptional activator and repressor complexes as a function of time after serum re-stimulation.

Based on the dynamics of protein-protein and promoter-protein interactions after mitogen stimulation, the authors demonstrate the consecutive formation of three distinct complexes centered around RUNX3. RUNX3 acts as a pioneer factor that binds specific target sites in closed chromatin and facilitates histone acetylation by p300. Subsequent recruitment of chromatin remodeling proteins via its binding

partner BRD2 generates an activator complex (AC) accumulating 2-4 h after mitogen stimulation that also includes MLL1/5 to produce the activating histone modification H3K4-me3. This results in transcriptional activation of RUNX3 target genes as exemplified by p14ARF; after 4-8 h another complex accumulates that contains PRC1 complex members RNF2 and BMI1 and also Cyclin D1 and HDAC4 (TR complex). At 8 h the RUNX3-BRD2 interaction is disrupted and PRC1 is replaced for PRC2 (repressor complex RE). This is associated with re-appearance of the repressive histone modification H3K27-me3 and loss of the activating modification H3K4-me3 as well as loss of ARF expression. Fig. 2f conveniently summarizes this interpretation although the inclusion of pRB in the early complexes is only justified later (Fig. 4a).

The picture that arises is quite complicated, but the authors have succeeded in presenting their data in clear figures and by lucid description in the manuscript text.

I only have some minor comments:

Comment:

1. Where does Cyclin D1 come from? The text reminds the reader that Cyclin D1 is transcriptionally and translationally controlled through RAS-RAF and RAS-PI3K pathways, respectively. Apparently, the raise of CYCLIN D1 levels occurs independently of RUNX3, but it would be convenient if the authors could refer to existing literature on CCND1 regulation.

We previously showed that the induction of Cyclin D1 is independent of RUNX3, as demonstrated by the fact that knockdown of RUNX3 does not affect serum-stimulated induction of CyclinD1 induction (Lee et al., Cancer Cell, 2013, Fig. 4D). We described this previous observation in the revised manuscript as follows: "Cyclin D1 is induced 2 hr after serum stimulation, and the induction of Cyclin D1 is independent of RUNX3 (Lee et al., Cancer Cell, 2013)." (page 8, lines 34-35).

Comment:

2. Related to the above: what is the rationale behind the early induction of p14ARF; why doesn't it stay repressed; why does ARF need to be opened by

RUNX3, activated and then again suppressed? Is it to provide the cells with p53 levels?

Answer:

Our results show that ARF is transiently induced during RAS is activated. The ARF–p53 pathway plays key roles in senescence/apoptosis; therefore, as the reviewer's question, the parallel levels of ARF expression and RAS activity appears to be meaningless or even harmful in normal cells. However, this phenomenon could provide a defense mechanism against oncogenic RAS. The oncogenic RAS mutation alters only the duration of the protein's activity, but not its maximum level of activity. Therefore, to defend against oncogenic RAS, cells should be able to measure the duration of RAS activity. Our results show that ARF is programmed to be expressed only when RAS is activated. When RAS is activated normally and downregulated soon thereafter, the ARF–p53 pathway should also be activated only transiently, and then suppressed, allowing cell cycle progression. On the other hand, if RAS is constitutively activated, the ARF–p53 pathway should also be constitutively activated, leading to senescence/apoptosis. This simple program enables cells to automatically distinguish oncogenic RAS from normal RAS. Our results suggest that opening, activation, and subsequent suppression of ARF by RUNX3 constitute a surveillance mechanism against oncogenic RAS.

Comment:

3. Most intriguing but also counterintuitive is that oncogenic RAS inhibits the transition of activator to repressor complexes and hence prevents the shut-down of cell cycle arrest genes like ARF. How does this work mechanistically? The data summarized in Fig. 5f implicates the RAS pathway at three levels into the AC-TR-RE transition. So how should the opposite activity of oncogenic RAS be interpreted?

Answer:

Although MEK, JNK, and PI3K signaling pathways are simultaneously activated by RAS, each pathway affects the Rpa-RX3-AC → Rpa-RX3-TR transition in a sequential manner. First, MEK signaling stimulates Rpa-RX3-AC formation 1 hr after serum stimulation. JNK signaling phosphorylates CDK4, which is recruited to Rpa-RX3-AC 2 hr later. PI3K signaling is delayed for more than 2 hr relative to MEK

signaling due to the requirement for *de novo* synthesis of Cyclin D1, a component of the PRC1/CyclinD1/HDAC4 complex that forms in response to PI3K signaling. This complex, in turn, induces the Rpa-RX3-AC → Rpa-RX3-TR transition. During the delay, MEK signaling is downregulated in normal cells. If MEK activity is not downregulated by expression of oncogenic RAS, PRC1/CyclinD1/HDAC4 complex formation is inhibited, leading to inhibition of the Rpa-RX3-AC → Rpa-RX3-TR transition. Therefore, the answer to the reviewer's question is that constitutively activated RAS signaling maintains Rpa-RX3-AC by inhibiting PRC1/CyclinD1/HDAC4 complex formation. We believe that this phenomenon constitutes an oncogene surveillance mechanism. To clarify this point, we included an overview of the differential regulations of the R-point by normal RAS and oncogenic RAS (Fig. 7f) in the revised manuscript.

Fig. 7f

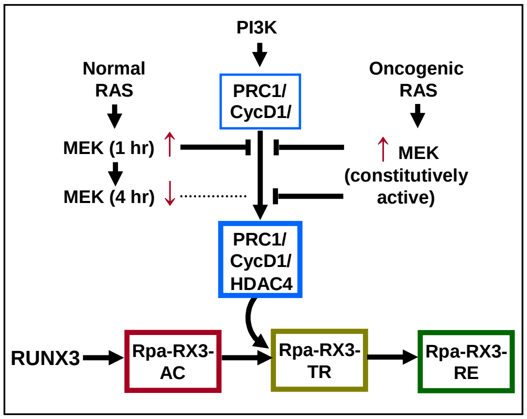

f Schematic illustration of differential regulation of the R-point in response to normal and oncogenic RAS. The RAS–RAF–MEK pathway inhibits formation of the PRC1/CyclinD1/HDAC4 complex, and thus inhibits the Rpa-RX3-AC → Rpa-RX3-TR transition. When the RAS–RAF–MEK pathway is activated by mitogenic stimulation, the activated pathway is downregulated 4 hr later, allowing formation of the PRC1/CyclinD1/HDAC4 complex formation, which is followed by the Rpa-RX3-AC → Rpa-RX3-TR transition. If the RAF–MEK pathway is activated by oncogenic RAS, the constitutively activated signal inhibits formation of the PRC1/CyclinD1/HDAC4 complex for a long period, thus inhibiting the Rpa-RX3-AC → Rpa-RX3-TR transition.

Comment:

4. The maintenance of the activator complex as a consequence of oncogenic RAS is presented as a protective mechanism against oncogenic transformation as it leads to sustained ARF levels and hence sustained p53. Indeed, H460 cells carrying an oncogenic K-RAS mutation, undergo p53-dependent apoptosis upon induction of RUNX3 expression. Have the authors looked at which cell cycle phase the cells entered apoptosis? Does the maintenance of the AC complex keep cells stuck at the restriction point, as the manuscript seems to suggest. Or are cells capable of entering S-phase.

Answer:

To answer the reviewer's question, we analyzed the cell-cycle stage of H460-ER-RX3 cells after 4-OHT treatment. The results revealed that about 49.7% and 13.1% of synchronized cells by serum starvation were at G1 and S stage, respectively, before 4-OHT treatment. Sixteen hours later, when the cells began to undergo apoptosis, the population of G1-phase cells increased slightly, whereas the abundance of S-phase cells decreased (G1=55.3% and S=9.33%). These results suggest that 4-OHT–treated H460-ER-RX3 cells entered apoptosis from the G1 phase without entering the S phase. We show these results in Supplementary Figure 10 and describe them in the revised manuscript as follows: "Analysis of cell-cycle stage of H460-ER-RX3 cells after 4-OHT treatment revealed that the populations of G1- and S-phase cells were slightly increased and decreased, respectively, by treatment with 4-OHT (Suppl. Fig. 10). These results suggest that 4-OHT–treated H460-ER-RX3 cells enter the apoptotic pathway from G1 phase without entering S phase." (page 15, line 11-15). The results and figure legend are shown below.

Supplementary Fig. 10

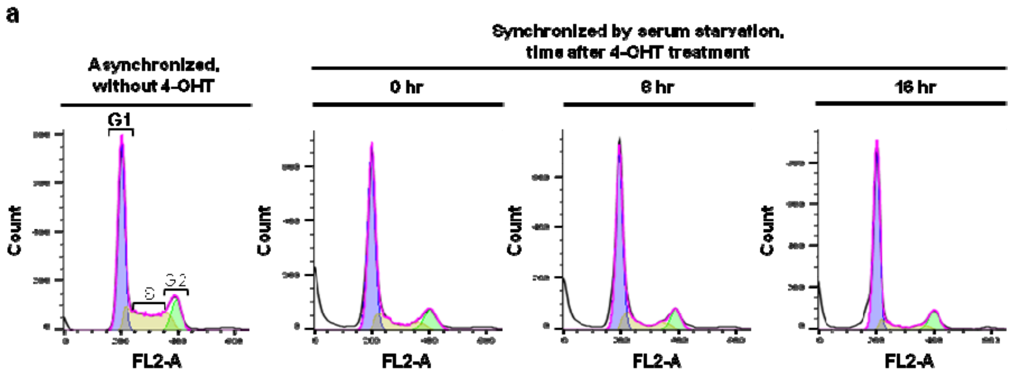

b

	Asynchronized	Synchronized, time after 4-OHT treatment		
	Con	0 hr	8 hr	16 hr
G1	48.9%	49.7%	51.5%	55.3%
S	28.4%	13.1%	12.6%	9.33%
C2	11.5%	8.75%	8.16%	3.[illegible]7%

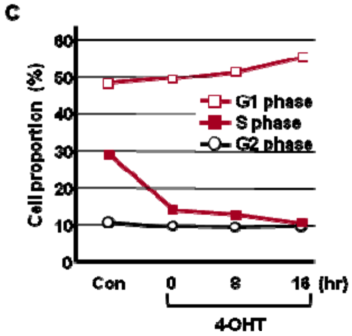

Supplementary Fig. 10. Analysis of cell-cycle stage in H460-ER-RX3 cells after 4-OHT treatment.

a H460-ER-RX3 cells were treated with 4-OHT, and populations of the cells were analyzed by FACS at various cell-cycle stages.

b Populations of H460-ER-RX3 cells after 4-OHT treatment are summarized.

c Changes in the cell-cycle stage populations of H460-ER-RX3 cells after 4-OHT treatment.

Comment:

5. In relation to the above: how do the authors relate their finding to earlier findings that oncogenic RAS drives cells prematurely into S-phase, which leads to replication stress, DNA damage and p53-dependent oncogene-induced senescence/apoptosis? Would this indicate that inhibition or abrogation of the AC complex to RE complex transition does not preclude S-phase entry? This also raises the question whether this transition is really instrumental for restriction point passage. Experiments to study effects on S-phase entry may provide more

insight.

Answer:

As the reviewer points out, expression of oncogenic RAS drives cells prematurely into S phase, leading to p53-dependent oncogene-induced senescence/apoptosis. However, the cellular responses to oncogenic RAS are slightly different depending on cell types. For example, expression of oncogenic *Ras* in primary human or rodent cells results in G1 arrest associated with a cell-cycle profile indistinguishable from that of serum-starved control cells. Arrest induced by *Ras* is accompanied by accumulation of p53 (Serrano et al., Cell. 1997; 88: 593-602). In this revised manuscript, we show that 4-OHT–treated H460-ER-RX3 cells entered the apoptotic pathway from the G_1 phase without entering S phase (Suppl. Fig. 10). Our results are consistent with those published by Serrano et al.: expression of oncogenic RAS inhibits cell-cycle progression at the R-point (early G1) by maintaining Rpa-RX3-Ac, which prolongs p53 expression.

Reviewer #3 (Remarks to the Author):

The authors have investigated the role of pioneer transcription factor RUNX3 in the restriction point. They found that there is a RUNX3-BRD2 complex that mediates changes in epigenetic factors through the sequential formation of two different protein complexes, affects gene expression, regulates the R point, and protects against oncogenes. The complexes include cyclin D1 and CDK4 and demonstrate a new role for this complex in cell cycle control. This interesting manuscript provides important new insights into the restriction point and how it is controlled. The data are clear and strong largely support the author's claims.

Comments

Supplementary Fig 1b: On what basis did the authors select these genes as R point genes?

Answer:

In Supplementary Fig. 1b, we showed transient induction of three R-point genes: p21, ARF, and p53. p21 was selected because the gene is induced 1–2 hr after serum stimulation in MEFs, and the phenomenon is associated with the R-point (Chi et

al., Oncogene. 2017; 36(50): 6884-6894). ARF and p53 were selected because the ARF–p53 pathway is transiently induced 1–2 hr after serum stimulation in HEK293 cells (Lee et al., Cancer Cell. 2013; 24(5): 603-16). We included references in the following sentence. "Consistently, expression of R-point–associated proteins [p14^{ARF} (hereafter ARF), p53, and p21] (Chi et al., Lee et al) was induced at the same time that RUNX3 interacted with BRD2, SWI/SNF, and TFIID (Suppl. Fig. 1b). " (page 5, lines 9-12).

Comment:

Supplementary Figure 1c. What is the 6KR mutant that the authors are using here?

Answer:

We did not describe the 6KR mutant construct in the original manuscript. In the revised manuscript, we described it as follows: "Myc-RX3-6KR mutant harbors K (Lys) to R (Arg) mutations in all 6 K residues within the Runt domain of RUNX3 (Figure legends in Suppl. Fig. 1c)."

Comment:

Supplementary Figure 1c. It is true that only when RX3 interacts with BRD2, it also interacts with TAF1, but these results may not be sufficient to conclude that interaction with BRD2 is required for TAF1 interaction as stated in the text.

Answer:

We showed that the RUNX3–TAF1 interaction is markedly reduced by knockdown of BRD2 (Fig. 1d). Based on this observation, we concluded that RUNX3 interacts with TAF1 through BRD2. The results are shown below.

Fig. 1d

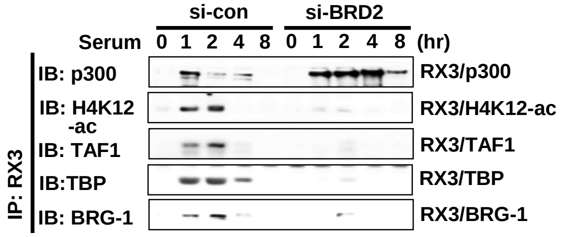

Comment:

Supplementary Figure 1c. Why does the RX3 mutant with 94-171 deleted run to the same size as the wild type RX3? Shouldn't it be smaller?

Answer:

We did not describe this mutant construct, previously described by Lee et al. (Cancer Cell, 2013), in the original manuscript. The Myc-RUNX3-KR-94-171 mutant is not a deletion; instead, the construct bears two point mutations at the indicated residues (K94R and K171R). Therefore, the 94–171 mutant protein is the same size as WT RUNX3. To avoid confusion, we changed the name of the mutant RUNX3 to Myc-RUNX3-K94/171R and described the construct in the revised manuscript as follows: "The Myc-RX3-K94/171R mutant, which does not interact with BRD2, harbors K94R and K171R mutations within the Runt domain of RUNX3 (Figure legends in Suppl. Fig. 1c)."

Suppl. Fig. 1c

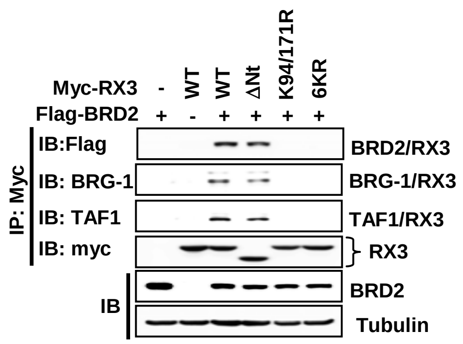

c HEK293 cells were transfected with *Myc-RUNX3-WT*, *Myc-RUNX3-ΔNt* (lacking N-terminal aa 1–54 region), *Myc-RUNX3-K94/171R* , *Myc-RUNX3-6KR*, or *Flag-BRD2-WT*, as indicated. Myc-RX3-6KR mutant harbors K (Lys) to R (Arg) mutations in all 6 K residues within Runt domain of RUNX3. The Myc-RUNX3-K94/171R mutant, which does not interact with BRD2, harbors K94R and K171R mutations within the Runt domain of RUNX3. Cells were serum-starved for 24 hr, and then stimulated with 10% serum. Cells were harvested 2 hr after stimulation, and the interactions of RUNX3 mutants with BRD2, BRG-1 (SWI/SNF), and TAF1 (TFIID) were measured by IP and IB. BRG-1 and TAF1 interacted with RUNX3 only when the RUNX3–BRD2 complex was formed.

Comment:

Fig 1e: The authors show that these deletion mutants are expressed at similar levels as wild type, but it is also important to know that the proteins encoded by these deletion mutants are localized to the nucleus similarly to wild type. The deletions could affect their nuclear localization.

Answer:

In response to the reviewer's comment, we analyzed subcellular localization of the deletion mutants shown in Figure 1e. The results revealed that all of the deletion mutants localized to the nucleus. We included these results in Supplementary Figure 1g and described them in the revised manuscript as follows: "Analysis of subcellular localization revealed that all of the BRD2 deletion mutants localized to the nucleus (Suppl. Fig. 1g)." The results and figure legend are shown below.

Suppl. Fig. 1g

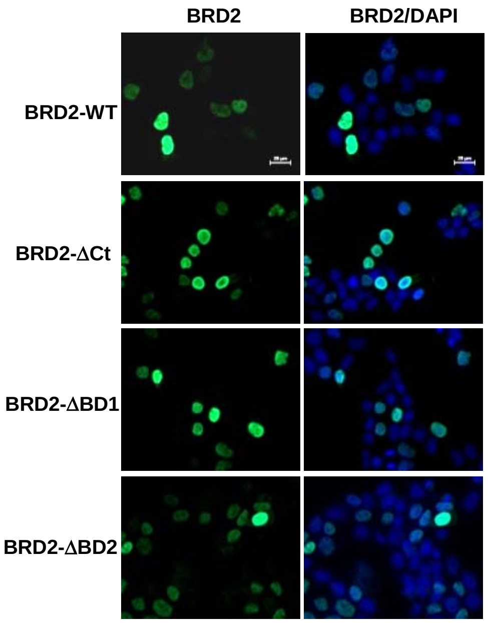

g HEK293 cells were transfected with *Flag-BRD2-WT*, *Flag-BRD2-ΔCt*, *Flag-BRD2-ΔBD1*, or *Flag-BRD2-ΔBD2*, as indicated. Subcellular localization of the expressed proteins was analyzed by immunofluorescence staining (green=BRD mutants; blue=DAPI).

Comment:

Fig 1e. The authors state that the result of these experiments will tell them the temporal order of events, but all samples are taken at the 2 hour time point. They clearly and convincing show the dependencies of the interactions. They show that RX3 can interact with p300 even in the absence of BRD2, and that BD1 and BD2 subdomains of RX3 are required for binding to H4K12-ac. But these results are not sufficient to draw conclusions about the temporal order of binding. In particular, I don't believe these results from a single time point can prove that BRD2 simultaneously binds acetylated RUNX3 and acetylated H4 through BD1 and BD2.

Answer:

The idea that BRD2 interacts simultaneously with acetylated RUNX3 and acetylated H4 through BD1 and BD2 does not originate from Figure 2e. Instead, we based this conclusion on the results shown in Figure 1b and 1d. In the original manuscript, we stated, "we detected interactions between p300, RUNX3, and H4K12-ac 1–2 hr after mitogenic stimulation (Fig. 1b, RX3/p300, RX3/H4K12-ac), as well as between BRD2, RUNX3, and H4K12-ac (Fig. 1b, BRD2/RX3 and BRD2/H4K12-ac). The RUNX3–H4K12-ac interaction was markedly diminished by knockdown of *BRD2* (Fig. 1d). These results suggest that RUNX3 guides p300 to target loci, where it acetylates histones, and that BRD2 binds both acetylated RUNX3 and acetylated histones through its two bromodomains prior to the R-point."

Fig. 1b

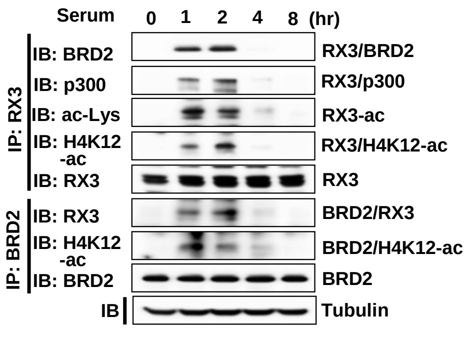

Fig. 1d

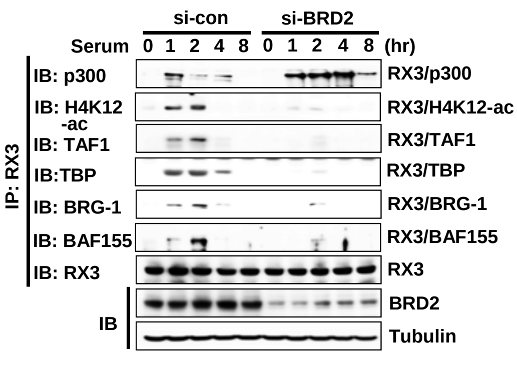

Figure 1e was provided to determine whether the SWI/SNF and TFIID complexes are recruited to BRD2 before or after formation of the RUNX3/BRD2/H4K12 complex. If SWI/SNF and TFIID complexes are recruited to

BRD2 after RUNX3/BRD2/H4K12 complex formation, deletion of BD1 or BD2 should abolish recruitment of SWI/SNF and TFIID. The results revealed that deletion of BD1 or BD2 from BRD2 indeed abolished recruitment of the SWI/SNF and TFIID complexes to BRD2 2 hr after serum stimulation. Therefore, we concluded that the SWI/SNF and TFIID complexes are recruited to BRD2 after the RUNX3/BRD2/H4K12 complex is formed.

Comment:

Fig 1f. This is a very dramatic result. Can the authors rule out the possibility that other places in the genome were also targeted by this Crispr guide in addition to the region upstream of p14? Are all of these proteins properly expressed and localized in the HEK293-ARF-RX-D mutant cells? Is it possible there are other proteins besides RUNX3 that also bind to this region of the p14 promoter?

Answer:

The ARF promoter contains four E2F-binding sites between ntd -300 and +50 from the transcription initiation site (Robertson and Jones, Mol Cell Biol. 1998; 18(11): 6457-73). To confirm that only the RUNX-binding sites were disrupted by our CRISPR engineering, we sequenced the region from ntd -2000 to +50 in HEK293-ARF-RX-D cells. The results confirmed that only the RUNX-binding site, and no other mutation, had been introduced into the ARF promoter region. We described these results in Experimental Procedures as follows: "Deletion of only the RUNX3-binding site in the ARF promoter region was confirmed by nucleotide sequencing." (page 23, lines 13-14). We included a short nucleotide sequence illustrating this point in Figure 1f, as follows:

Fig. 1f

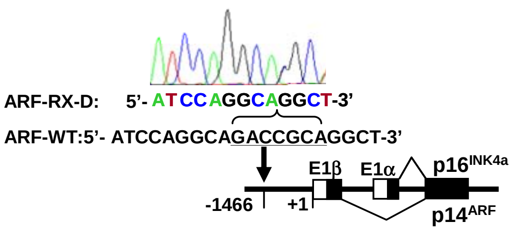

Comment:

Fig 1g. pRB is present in the figure at 2 hours but the authors do not present data showing that pRB is part of this complex.

Answer:

We included pRB as a component of Rpa-RX3-AC in Figure 2f because we previously showed that the pRB/RUNX3/BRD2 complex is formed 1–2 hr after serum stimulation, and that the complex contributes to R-point regulation (Chi et al., Oncogene. 2017; 36(50): 6884-6894). In this paper, we showed that E2F1 as well as pRB (Chi et al., Oncogene. 2017; 36(50): 6884-6894) is a component of Rpa-RX3-AC (Fig. 4a). To avoid confusion, we erased pRB from Figure 2f and included it in Figure 4f as a component of Rpa-RX3-AC.

Comment:

Fig 2f: Because the data points were actually taken at 2, 4 and 8 hours, it seems more appropriate for the authors to draw models for 2, 4 and 8 hours rather than 3 hours. RNF2 seems particularly important in distinguishing 2 and 4 hours. Can it be added to the model?

Answer:

As the reviewer suggested, we provided models for 2, 4, and 8 hr in Figure 2f. The new schematics are shown below.

Fig. 2f

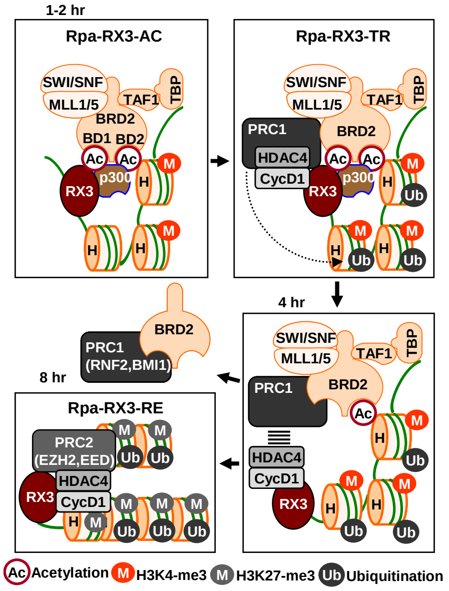

Comment:

Line 259: The data in figure 2e do not demonstrate simultaneous recruitment to target loci.

Answer:

We agree. We changed the sentence as follows: "PRC1, CyclinD1, and HDAC4 were recruited to the target loci 4 hr after serum stimulation (Fig. 2e)." (page 9, lines 14-15).

Comment:

Fig 5 and line 339-342: The authors show very convincingly that JNK is required for RUNX3 phosphorylation and for the dissociation of RUNX3 from BRD2 and CDK4. Are the authors arguing that phosphorylation of RX3 is mediated by CDK4 that has been phosphorylated by JNK but without any cyclin bound? CDKs do not have a good binding pocket for target proteins when cyclin is not bound so this model is surprising. Cyclin D1 levels may not have peaked at 2 hours, but it is still possible that the CDK molecules performing the phosphorylation are associated with cyclin D1 (or another cyclin D). Indeed, Fig 5d and 5e seem to suggest cyclin D1 itself is important for the dissociation of BRD2 and RX3.

Answer:

We agree that the possibility remains that RUNX3 is more susceptible to CDK4/CyclinD1-mediated phosphorylation than pRB, and that a very low level of CDK4/CyclinD1 complex could be responsible for RUNX3 phosphorylation. To investigate this possibility, we knocked down Cyclin D1 and measured phosphorylation of CDK4-mediated pRB and RUNX3. The results revealed that knockdown of Cyclin D1 abolished pRB phosphorylation (S795), but did not affect RUNX3 phosphorylation (S356). These results support our claim that CyclinD1 is not involved in CDK4-mediated RUNX3 phosphorylation (S356). We included these results in the revised manuscript (Fig. 5b) and described them as follows: "Knockdown of Cyclin D1 effectively reduced pRB phosphorylation (S795), but did not affect RUNX3 phosphorylation (S356) (Fig. 5b). These results suggest that CDK4 is activated in a CyclinD1-independent manner to promote RUNX3 phosphorylation." (page 12, lines 3-6). The results are shown below:

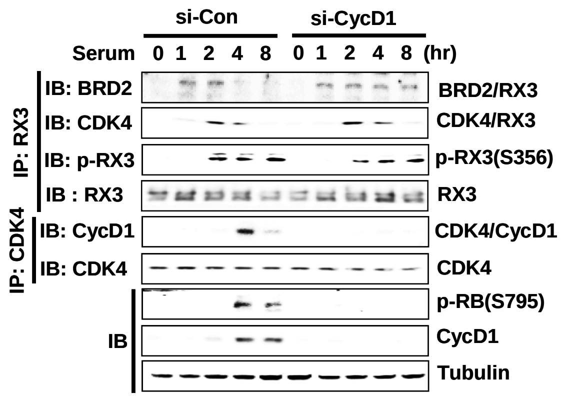

b HEK293 cells were treated with control or CyclinD1-specific siRNA (si-con or si-CycD1), serum-starved for 24 hr, and then stimulated with serum for the indicated durations. Time-dependent formation of the BRD2–RUNX3 and CDK4–RUNX3 complexes and phosphorylation of RUNX3 at Ser-356 and pRB at Ser-795 were measured by IP and IB.

> Indeed, Fig 5d and 5e seem to suggest CyclinD1 itself is important for the dissociation of BRD2 and RX3.

As the reviewer commented, Figure 5d and 5e and the figure shown above (knockdown of CyclinD1) also demonstrate the critical role of CyclinD1 in the dissociation of BRD2 and RX3. However, these results do not mean that CyclinD1 is involved in CDK4-mediated RUNX3 phosphorylation, because knockdown of CyclinD1 maintained the BRD2–RUNX3 interaction but did not affect RUNX3 phosphorylation (Fig. 5b).

Comment:

Fig 7: H460 cells do not normally apoptose and they presumable have activated RAS and endogenous RX3. Why is it that introducing RX3 with an estrogen receptor and adding tamoxifen results in apoptosis?

Answer:

In H460 cells, K-RAS is activated and RUNX3 is silenced. This is described in the manuscript as follows: "We next investigated the protective role of RUNX3 against endogenous oncogenic *K-RAS* in H460 human lung cancer cells (*K-RAS*–activated without amplification, *RUNX3*-inactivated, *ARF* wild-type, and p53 wild-type)." (page

14, lines 19-21). To analyze the role of RUNX3 in oncogenic K-RAS–induced ARF–p53 pathway activation, we introduced RUNX3-estrogen receptor in the H460 cells, and then treated with tamoxifen. Because introduction of RUNX3 induces apoptosis, we could not obtain a H460 cell line stably expressing RUNX3. Therefore, RUNX3 was fused with the estrogen receptor to make it inducible.

Comment:

Fig 7e,f: Related to above, if the ER-RX3 cells are apoptosing in response to 4 OHT treatment, but H460 cells do not normally spontaneously apoptose, this may not be the best conditions for determining the downstream targets of RUNX3.

RUNX3 induces apoptosis in cells expressing oncogenic K-RAS, but not in normal cells. In H460 cells, K-RAS is activated and RUNX3 is silenced. When ER-RUNX3 is activated by 4-OHT, RUNX3 forms Rpa-RX3-AC 8 hr later (Fig. 8b) and the cells undergo apoptosis at 16 hr (Fig. 8d). Therefore, we can investigate downstream targets of RUNX3 8 hr after 4-OHT treatment. Based on this logic, we believe that the H460-ER-RX3 cell line is a good model for determining the downstream targets of RUNX3 that induce oncogenic K-RAS–dependent apoptosis.

REVIEWERS' COMMENTS:

Reviewer #1 (Remarks to the Author):

The revised manuscript "RUNX3 regulates cell cycle-dependent chromatin dynamics by functioning as a pioneer factor of the restriction point" addresses thoroughly most of comments from the different reviewers. Therefore, while the original manuscript was already very interesting and of good quality, this new version is definitely strengthen and well suited for publication in Nature Communications journal.

I only have minor comments/questions:
- in different figures regarding WB detection of H4ac, I am surprised by the size shown for H4 (<7kDa).
- how representative is coIP RX3/p300 from fig1b and fig1d(siControl)? indeed, the result and timing of interaction is quite different in both panel. Even more disturbing is the increase of RX3/p300 interaction
- regarding Fig1, there is no reason to show CycD1 & HDAC4 at this point as there is no mention of it in the manuscript.
- while authors nicely tested RX3 complex formation after EGF and doxorubicin (suppl fig1F, why do they only look at 12h and 24h after doxorubicin and not earlier like for every other experiment (1 to 8h).
- In Fig7f, the claim that MEK1 (or more likely phosphoMEK1) is increasing at 1h but decreases at 4h while not changing in oncogenic RAS mutant cells is not backed up by WB data. Can authors provide this confirmation?

All my other comments have been clearly answered by authors in rebuttal, and I have no other concern regarding this excellent article.

Reviewer #2 (Remarks to the Author):

The authors have meticulously and to my taste satisfactorily addressed the comments raised by the reviewers, performed additional experiments to clarify several issues and adapted their manuscript accordingly.

Only one small point is unclear:

In an attempt to determine whether induction of RUNX3 expression in K-RAS mutant H460 cells allows cells to enter S-phase, the authors have performed a cell cycle analysis. The outcome of the experiment, presented in Supplementary Fig. 10 , shows a decrease in S-phase cells and a slight increase in G1 cells. The level of S-phase decrease is somewhat unclear: the figure shows a decline from 28.4% to 9.33 %; the text says "slightly decreased"; the rebuttal letter mentions: from 13.1% to 9.33%. Whatever the precise figure is, the authors conclude that "..cells enter the apoptotic pathway from G1 phase without entering S phase". However, couldn't apoptosis in S-phase be the cause of the loss of S-phase cells rather than reduced S-phase entry as the authors propose?

In their rebuttal, the authors consider their work supporting Serrano et al., 1997, who postulated Ras-induced cell cycle arrest to result from direct accumulation of p53. Later work however suggested involvement of replicative DNA damage. I do appreciate the attempt the authors have made to clarify this point and leave it to them to interpret the result as they did. However, in my opinion the debate on the possible involvement of DNA damage has not been closed.

Reviewer #3 (Remarks to the Author):

The authors addressed my questions. I have no other comments.
